# *Fridericia chica* standardized extract combined with tocotrienol-enriched fraction from *Bixa orellana* promotes gastric mucosal protection: *in vitro* combination selection and *in vivo* evaluation

**DOI:** 10.1080/13880209.2026.2751273

**Published:** 2026-10-24

**Authors:** Ailane Souza de Freitas, Ilza Maria de Oliveira Sousa, Fabricio de Faveri Favero, Josélia Cristina de Oliveira, Robson da Silva Pontes, Alex Vinicius de Campos, Clara Giuliana Magossi, Mayra Gabriella Biccigo, Ana Lucia Tasca Gois Ruiz, João Ernesto de Carvalho, Mary Ann Foglio

**Affiliations:** School of Pharmaceutical Sciences (FCF), Universidade Estadual de Campinas (UNICAMP), Campinas, Brazil

**Keywords:** *Arrabidaea chica*, combination, gastric ulcer, vitamin E, anthocyanins

## Abstract

**Context:**

*Fridericia chica* (Bonpl.) L.G. Lohmann is a medicinal plant rich in bioactive 3-deoxyanthocyanidins with reported wound-healing and gastroprotective properties. A 70% tocotrienol-enriched fraction (TRF) from *Bixa orellana* L. exhibits antioxidant and anti-inflammatory activities that may complement those of *F. chica* in gastric mucosal protection.

**Objective:**

This study aimed to evaluate the gastroprotective and gastric tissue-repair effects of the *F. chica*–TRF combination.

**Materials and methods:**

Dry *F. chica* standardized crude extract alone or combined with TRF at different proportions was tested *in vitro* in HaCaT keratinocytes and 3T3 fibroblasts to assess cell viability and select a suitable combination ratio for subsequent *in vivo* investigation. The selected ratio was then investigated in acute and chronic gastric ulcer models in female Wistar rats.

**Results:**

Among the proportions tested *in vitro*, the 90:10 (*F. chica*:TRF) ratio showed the most favorable cell-viability profile and was selected for *in vivo* evaluation. The combination reduced gastric lesions and significantly increased gastric mucus production. In the chronic ulcer model, treatment was associated with reduced TNF-*α* and IL-1*β* levels and increased IL-10 levels. Histopathological and second-harmonic generation analyses qualitatively indicated mucosal preservation, tissue organization, and collagen deposition.

**Conclusions:**

The *F. chica*–TRF combination exhibited gastroprotective effects associated with increased mucus production and modulation of inflammatory mediators. Qualitative morphological findings further indicated preservation of gastric tissue architecture and collagen organization, supporting the potential of this combination as a natural product-based approach to gastric mucosal protection.

## Background

Gastric ulcers are among the most common diseases of the digestive system worldwide characterized by mucosal erosion and gastric defects (An et al. 2026; Shu et al. 2026). Epidemiological data show that between 1990 and 2019, the global incidence rate of peptic ulcers, which includes gastric and duodenal ulcers, increased by 27.3%. In 2019, the global prevalence was approximately 8.09 million cases, representing a 25.82% increase compared to 1990 (Xie et al. 2022).

The etiology of gastric ulcers is multifactorial and involves an imbalance between mucosal protective and aggressive agents (Ribeiro et al. 2026). Protective agents include the controlled production of mucus, prostaglandins, nitric oxide, bicarbonate, growth factors, the presence of endogenous antioxidants, and adequate blood flow. Endogenous aggressive factors include excessive production of hydrochloric acid and pepsin, biliary reflux, reactive oxygen species (ROS), reduced blood flow, and *Helicobacter pylori* infection (Mertz and Walsh 1991; Xie et al. 2022).

A meta-analysis of clinical and epidemiological studies conducted between 1990 and 1995 identified non-steroidal anti-inflammatory drugs (NSAIDs) use, *Helicobacter pylori* infection, and smoking as the main risk factors, accounting for 89% to 95% of ulcer cases during that period (Kurata and Nogawa 1997). Since the 2000s, there has been a growing trend in NSAIDs use, especially in patients over 60 years of age. In addition to these exogenous aggressive agents, stress, poor diet, alcohol consumption, and other aspects of the modern lifestyle have strongly contributed to the global prevalence of gastric ulcers, generating a greater need for new effective therapeutic agents (Xie et al. 2022).

In the 1970s, the introduction of cimetidine, a histamine H_2_-receptor antagonist, represented a significant advance in the treatment of gastric ulcers by inhibiting histamine-mediated acid secretion. Subsequently, in 1982, omeprazole was developed, a proton pump inhibitor (H^+^/K^+^-ATPase) that acts directly at the final step of acid secretion (Toneto et al. 2011).

However, the use of these medications has been associated with serious adverse effects. Proton pump inhibitors, for example, are associated with an increased risk of fractures, kidney disease, infections, and nutritional deficiencies of calcium, magnesium, and vitamin B12, in addition to amplifying NSAIDs-induced lesions and a higher risk of gastric cancer. Ranitidine, on the other hand, was withdrawn in several countries following the detection of the carcinogenic substance N-nitrosodimethylamine (NDMA) (Abu-Baih et al. 2024).

In this context, the search for low-toxicity, easily accessible, and low-cost medications has sparked interest in traditional medicine and the study of natural products as sources of new drugs.

*Fridericia chica* (Bonpl.) L.G. Lohmann (syn. *Arrabidaea chica* Verlot*)* is a shrubby plant found in tropical American countries such as Brazil, Costa Rica, Paraguay, and French Guiana, and less frequently in the United States and New Zealand. In Brazil, this species is popularly known as *crajiru* or *pariri* and is widely distributed, occurring in all regions and phytogeographic domains (GBIF 2023).

Previous studies have demonstrated the plant’s healing activity on external wounds and gastric ulcers (Figueiredo et al. 2023; Miorando et al. 2025; Sousa et al. 2025). In a phase II/III clinical trial, participants with onco-hematological diseases undergoing bone marrow transplants were randomized to treat mucositis with a 2.5% *F. chica* mucoadhesive gel or low-level laser therapy. Results showed that the mean healing time for mucositis was 5.2 ± 1.5 days in the intervention group and 11.2 ± 6.5 days in the control group, representing 2.2 times faster healing with the gel containing the standardized crude extract of *F. chica* (Queiroz et al. 2025).

This healing effect has been attributed to the 3-deoxyanthocyanidins 6,7,3,4-tetrahydroxy-5-methoxy-flavylium, 6,7,4′-trihydroxy-5-methoxyflavylium, and 6,7-dihydroxy-5,4-dimethoxy-flavylium, a select group of flavonoids present in the plant, recognized for their antioxidant capacity (Sousa et al. 2025).

Due to its antioxidant and anti-inflammatory properties, the 70% tocotrienol-enriched fraction (TRF), a byproduct of bixin (*Bixa orellana* L.) production, was investigated in combination with standardized *F. chica* extract based on the distinct pharmacological properties of these natural products. Although the gastroprotective effects of *F. chica* and tocotrienols have been individually investigated, the effects of their combination on gastric mucosal protection and tissue repair have not yet been established. In addition, an appropriate proportion of these components that preserves cell viability for subsequent *in vivo* evaluation has not been defined. Therefore, we hypothesized that a *F. chica*–TRF combination selected on the basis of *in vitro* cell viability would exert gastroprotective effects and modulate parameters associated with gastric mucosal protection and tissue repair in experimental ulcer models. Accordingly, this study aimed to select an appropriate *F. chica*–TRF proportion based on *in vitro* cell viability and subsequently evaluate its effects in acute and chronic experimental models of gastric ulcer.

## Material and methods

### Plant material

*F. chica* leaves were collected from the Germplasm Bank, accession 06 (voucher specimen 1865), at the Multidisciplinary Center for Chemical, Biological, and Agricultural Research (CPQBA—UNICAMP) experimental field, located in Paulínia, SP, Brazil (22°45′00″S, 47°10′21″W), in the morning during March 2021. The voucher was identified by Professor Maria do Carmo Estanislau do Amaral from the Botany department at Biology Institute at Unicamp.

The extraction protocol employed in the present study followed a standardized procedure previously developed and consistently adopted by our research group to produce *F. chica* extracts. This protocol includes the use of accession 06, standardized cultivation and harvesting conditions, extraction with acidified hydroethanolic solvent, and spray-drying processing (Figueira et al. 2010; Paula et al. 2013; Taffarello et al. 2013; Servat-Medina et al. 2015; Zago 2020; Queiroz et al. 2025; Sousa et al. 2025). The adoption of this standardized protocol has enabled the reproducible production of *F. chica* extracts for phytochemical, preclinical, pharmaceutical, and clinical studies conducted by our research group.

The leaves were dried for 48 h in a forced-ventilation oven at 40 °C (Fabbe^®^) and subsequently ground in a hammer mill (Stephen^®^ UM 40) using a 40-mesh sieve. The dry, ground material was transferred to a 50 L stainless steel tank with mechanical stirring. The extraction solvent used was 70% ethanol (Dinâmica^®^—Cat. No. P.01.0024.006.06.81) (acidified with 0.3% citric acid (Dinâmica^®^—Cat. No. P.10.0021.042.03.30), at a ratio of 1:5 w/v (plant:solvent). This process was repeated three times, each lasting approximately 90 min. The resulting extract was concentrated under vacuum using a rotary evaporator (Büchi^®^ R-200) to remove ethanol, achieving an 80% reduction of the initial volume.

A mini spray dryer (Haurok^®^ SD-1.8L) was used to dry the aqueous extract under the following parameters: inlet temperature of 140 °C, air inlet at 70%, feed speed at 15%, air inlet flow of 7.5 L/min, and needle cleaning frequency of 500 s. The outlet temperature remained between 99 and 101 °C throughout the process. The dry material was stored in aluminum packaging in a freezer until use.

The tocotrienol-enriched fraction (TRF) was provided by Dr. Paulo Carvalho from the Institute of Food Technology (ITAL) in Campinas, SP, and was obtained from *B. orellana* bixin dye production residue *via* a patented molecular distillation process (BR 102015029422-A2). According to a chemical characterization study conducted by our research group using the same TRF batch employed in the present study, the fraction contained 63.62 ± 1.34% *δ*-tocotrienol and 8.00 ± 0.21% *γ*-tocotrienol, corresponding to a total tocotrienol content of 71.62 ± 1.57% (Rabelo 2025).

Authorization to access genetic heritage for this study is registered in the National System for the Management of Genetic Heritage and Associated Traditional Knowledge (SisGen) under number A5489C9.

### Chromatographic analysis of *Fridericia chica* extract

The major 3-deoxyanthocyanidins of *F. chica*, including 3′-hydroxy-carajurone, carajurone, and carajurin, were previously identified and chemically characterized by nuclear magnetic resonance spectroscopy and mass spectrometry in studies conducted by our research group (Figueira et al. 2010; Taffarello et al. 2013; Servat-Medina et al. 2015; Sousa et al. 2025). These compounds have been consistently used as characteristic phytochemical markers of the standardized extract.

Carajurin was isolated from the standardized crude extract of *F. chica*, structurally identified, and used as a reference compound for its quantification in the extract. Commercial analytical standards of apigenin (LGC Standards^®^, Middlesex, UK; 96% purity. Cat No. ASB-00001900) and luteolin (Cayman Chemical^®^, Ann Arbor, MI, USA; 98% purity. Cat. No. 10004161) were used for the quantitative determination of these flavones, which are naturally present in *F. chica* and have been associated with its wound-healing activity.

Briefly, chromatographic analysis was performed using an HPLC system (Agilent^®^ 1100 Series, Agilent Technologies, Santa Clara, CA, USA) equipped with a UV/Vis detector and a C18 column (Gemini, 250 × 4.6 mm, 5 μm; Phenomenex^®^, Torrance, CA, USA). The mobile phase consisted of ultrapure water containing 0.1% formic acid (J.J. Baker^®^, Cat. No. 0128-02) and methanol (Dinâmica^®^, Cat. No. P.10.0051.018.0981), with constant flow rate of 1.0 mL min^−1^ under gradient elution. The gradient program was: 0–5 min, 65% A and 35% B; 5–25 min, 35% A and 65% B; 25–28 min, 30% A and 70% B; 28–35 min, 10% A and 90% B; and 35–45 min, 65% A and 35% B. The injection volume was 20 μL. Detection was performed at 470 nm for the identification of the characteristic 3-deoxyanthocyanidins of *F. chica*, namely 6,7,3′,4′-tetrahydroxy-5-methoxyflavylium (3′-hydroxy-carajurone), 6,7,4′-trihydroxy-5-methoxyflavylium (carajurone), and 6,7-dihydroxy-5,4′-dimethoxyflavylium (carajurin). The isolated carajurin was additionally used as the analytical reference standard for its quantitative determination. Apigenin and luteolin were detected and quantified at 375 nm. For quantitative analysis, three independently prepared sample solutions of the *F. chica* extract were analyzed under the same chromatographic conditions, and the results were expressed as the mean ± standard deviation (SD).

### *In vitro* pharmacological evaluation

#### Cell lines

Immortalized human keratinocyte cell lines (HaCaT, CVCL_0038, male, Caucasian, 62 years old) and immortalized murine fibroblasts (NIH 3T3, CVCL_0594; male Swiss mouse embryo) were kindly provided by Prof. Dr. Ricardo Della Coletta (Piracicaba Dental School, FOP-Unicamp) and Prof. Dr. Marcelo Lancellotti (Faculty of Pharmaceutical Sciences, FCF-Unicamp), respectively. Cells were cultured in 25 cm^2^ flasks and maintained at 37 °C in a humidified atmosphere with 5% CO_2_. The HaCaT cells were grown in RPMI 1640 medium (Gibco^®^—Cat. No. 31800-14) supplemented with 5% fetal bovine serum (FBS, Vitrocell^®^—Cat. No. S0011), while NIH 3T3 cells were cultured in Dulbecco’s Modified Eagle Medium (DMEM, Sigma-Aldrich^®^—Cat. No. D0822) supplemented with 10% (FBS). For the experiments, the complete medium was supplemented with 1% penicillin-streptomycin solution (10,000 U/ml: 10 mg/ml, Sigma-Aldrich^®^—Cat. No. P4333). All experiments were performed using cells between passages 6 and 10 after thawing.

#### Samples preparation

*F. chica* and TRF stock solutions were prepared in dimethyl sulfoxide (DMSO, Sigma-Aldrich^®^—Cat. No. D2653) and subsequently diluted in complete medium or medium supplemented with 0.2% FBS to obtain the desired final concentrations (0.2–100 µg/mL for *F. chica* and 0.09–50 µg/mL for TRF).

#### Cell viability evaluation

These experiments followed the protocols described by Monks et al. (1991) and Jorge et al. (2008) with minor modifications.

In the first experiment, a HaCaT cell suspension (4 × 10^4^ cells/mL) was distributed into two 96-well plates (100 µL/well), followed by a 24-h incubation. Thereafter, in plate T1, the medium was replaced with RPMI (Gibco^®^—Cat. No. 31800-14) containing 0.2% FBS (Vitrocell^®^—Cat. No. S0011), while plate T2 was maintained with complete medium and incubated for a further 24 h. Then, cells in plates T1 and T2 were exposed for 48 h to *F. chica* (0.2–100 µg/mL) or TRF (0.09–50 µg/mL).

In the second experiment, 3T3 and HaCaT cell suspensions (4 × 10^4^ cells/mL) were distributed into 96-well plates (100 µL/well), followed by a 24-h incubation. Then, cells were treated with *F. chica*, *F. chica*/TRF 90:10, *F. chica*/TRF 95:5, *F. chica*/TRF 99:1, or TRF (3–100 µg/mL).

Forty-eight hours after treatment in both experiments, cells were fixed with 50% trichloroacetic acid (50 µL/well, 1 h, 4 °C, Merck^®^—Cat. No. T4885) and stained with 0.4% sulforhodamine B (SRB, Sigma-Aldrich^®^—Cat. No. S1402) in 1% acetic acid (50 µL/well, 20 min, Labsynth^®^—Cat. No. A1019.01.BJ). Bound SRB was solubilized with Trizma^®^ base (10 mM, pH 10.5, Merck^®^—Cat. No. 1503) and the absorbance at 540 nm was measured with a microplate reader.

For cell viability evaluation, untreated cells after 48 h of incubation were considered to represent 100% of viability (Equation 1), and the effective concentration was expressed as the half-maximal inhibitory concentration (IC_50_). Concentration-response curves and IC_50_ values were determined using ORIGIN^®^ 8.0 software (OriginLab Corporation). For all assays, each concentration was evaluated in technical triplicate in two independent experiments.

(1) $$\text{Cell viability }(\%)=\frac{\text{TA }}{T1}\times 100$$

in which, TA is the mean absorbance of treated cells corrected for sample blank absorbance and T1 is the absorbance of the untreated cell suspension.

#### Combinatory analysis and isobologram

Based on the IC_50_ values obtained for HaCaT cells, a second concentration range (3, 10, 30, and 100 µg/mL) was selected for evaluation in both HaCaT and 3T3 cells. For the construction of isobolograms, individual IC_50_ values of *F. chica* and TRF were used to calculate the theoretical IC_50_ values for the combination ratios of 90:10, 95:5, and 99:1 (*F. chica*/TRF). Both theoretical and experimental IC_50_ values were then plotted in isobolograms using ORIGIN^®^ 8.0 software.

### *In vivo* pharmacological evaluation

#### Animals

One hundred and forty female Wistar rats were from the Multidisciplinary Center for Biological Investigation in Laboratory Animal Science of the University of Campinas (CEMIB-UNICAMP, Campinas, Brazil). Animals were adapted to vivarium conditions for at least 30 days prior to experimental procedures and maintained at 25 ± 2 °C under a controlled 12 h light–dark cycle. Ventilated racks housed rats provided with Puro Trato^®^ chow and water *ad libitum*. All experiments used adult animals aged 12 weeks.

A simple random allocation procedure based on cage selection was used to distribute the experimental animal groups. Upon arrival at the animal facility, the rats were randomly assigned to cages, housing six animals each, and acclimatized under standard laboratory conditions until the start of the experiments. Experimental procedures were performed under the same environmental conditions and at similar times of day to minimize potential confounding factors. Investigators were aware of group allocation during treatment administration and outcome assessment. To reduce subjectivity in the assessment of gastric lesions, stomachs were photographed under standardized experimental conditions, and lesion areas were quantitatively determined from the acquired images using ImageJ^®^ software. Other outcomes, including gastric mucus and cytokine levels, were determined using quantitative analytical methods.

General clinical condition was monitored throughout the *in vivo* experiments, with animals observed for overt signs of distress or treatment-related clinical alterations. In the chronic acetic acid-induced ulcer model, body weight was additionally monitored daily throughout the seven-day treatment period. Animals were euthanized by anesthetic overdose with ketamine hydrochloride 10% (300 mg/kg; Cetamin^®^, Syntec do Brasil Ltda., Santana de Parnaíba, SP, Brazil) and xylazine hydrochloride 2% (300 mg/kg; Xilazin^®^, Syntec do Brasil Ltda., Santana de Parnaíba, SP, Brazil), administered intraperitoneally, followed by cervical dislocation. Euthanasia was performed 2 h after ethanol-induced gastric ulcer induction, 4 h after gastric secretion quantification, and on the eighth day of the acetic acid-induced gastric ulcer protocol. All experimental procedures were approved by the Ethics Committee on Animal Use of the University of Campinas (CEUA/UNICAMP, protocol no. 6531-1/2024, approved on October 17, 2024) and were conducted between March 19 and May 24, 2025.

Sample size was determined a priori and approved by the institutional Animal Ethics Committee (CEUA/UNICAMP). The minimum number of animals required per group was calculated using the formula *n* = 1 + [2C(s/d)^2^], where C = (Zα + Zβ)^2^, s represents the maximum expected standard deviation, and d represents the expected difference between groups. Assuming a significance level of 95%, a maximum standard deviation of 20%, and an expected difference of 50% between groups, the calculated sample size was 5.97 animals per group. Therefore, six animals per group were included in the ethanol-induced gastric ulcer experiments. In the acetic acid-induced chronic gastric ulcer model, the same sample size was initially adopted; however, animals in which ulcer induction was unsuccessful, as evidenced by the absence of a gastric lesion or the presence of only a minimal lesion, were excluded from the final analysis according to predefined experimental criteria. Consequently, the final sample size analyzed in this experiment was four animals per group.

#### Sample preparation and treatment techniques

Aliquots of *F. chica* extract and TRF were dispersed in 0.9% NaCl solution (Dinâmica^®^—Cat. No. P.10.0294.078.00.27) containing 0.2% (w/v) carboxymethylcellulose (CMC; Sigma-Aldrich^®^—Cat. No. C4888) and mixed immediately prior to administration. The NaCl solution containing 0.2% CMC constituted the vehicle (saline). Carbenoxolone (Merck^®^—Cat. No. CA790) and cimetidine (Merck^®^—Cat. No. C4522) were prepared in the same vehicle immediately before use. The concentrations administered in each experiment were adjusted according to the corresponding experimental protocol. All oral treatments were administered by gavage using a flexible orogastric catheter (2 mm, 5 cm length). All intraperitoneal (i.p.) and intraduodenal treatments were administered using syringes (1 or 3 mL) fitted with needles (13 × 0.45 mm or 20 × 0.55 mm).

#### Ethanol-induced ulcer

Female Wistar rats (*n* = 60) were divided into experimental groups (*n* = 6 per group) and individually identified. Body weight was recorded one day before the experiment and fasted for 18 h.

For the dose–response curve assay, groups received orally: vehicle (saline, 2.5 mL/kg), positive control (carbenoxolone, 200 mg/kg, Merck^®^—Cat. No. CA790), or *F. chica* + TRF (30, 100, and 300 mg/kg; 90:10 ratio, defined from *in vitro* results). For ulcer evaluation, in addition to the vehicle and positive control groups, *F. chica* (135 mg/kg), TRF (15 mg/kg), and *F. chica* + TRF (ED_50_ = 150 mg/kg, selected based on the exploratory dose–response analysis) were included.

One hour after treatment, analytical-grade ethanol (4 mL/kg, p.o.; Halogenn, Brazil; Cat. No. HL100.006) was administered. Thereafter, one hour after lesion induction, animals were euthanized by anesthetic deepening (ketamine 300 mg/kg + xylazine 300 mg/kg) followed by cervical dislocation.

Stomachs were excised, opened along the greater curvature, and washed with 0.9% saline solution. Tissues were pressed between glass plates and photographed (Figueiredo et al. 2023).

The area of ulcerative lesions was determined using ImageJ^®^ software. The percentage of ulcerated area and the healing index were estimated according to the following equations. Small tissue fragments (*n* = 3/group) were collected for histological analysis using hematoxylin and eosin (H&E) and Masson’s trichrome staining.

(2) $$\text{\% Ulcerated area }=\frac{\text{sample area}\;}{\text{vehicle area}\;}\times 100$$

(3) $$\text{\% Healing }=\frac{\text{vehicle}\;\text{area}-\text{sample}\;\text{area}\;}{\text{vehicle}\;\text{area}}\times 100$$

### Gastric mucus quantification

Following macroscopic evaluation and image acquisition of stomachs from the ethanol-induced gastric ulcer model, the same tissues (*n* = 6 animals per group; total *n* = 30) were weighed and incubated in 0.1% alcian blue solution (10 mL, Merck^®^—Cat. No. A3157) for 2 h. Excess dye was then removed by successive washings with 0.25 M sucrose (Labsynth^®^—Cat. No. S.2609.01.AH).

The dye bound to the gastric mucus layer was extracted with 0.5 M MgCl_2_ (10 mL, (Labsynth^®^—Cat. No. C2014.01.AG) under intermittent agitation for 2 h. Aliquots of the supernatant (5 mL) were mixed with ethyl ether (1:1, v/v, Ecibra^®^—Cat. No. 765), shaken, and centrifuged (3600 rpm, 15 min). The organic phase was discarded, and the aqueous phase was used for spectrophotometric reading at 598 nm.

The amount of mucus was determined using an alcian blue standard curve (6.25–100 µg/mL), with results expressed as µg of alcian blue/g of tissue (Figueiredo et al. 2023).

### Gastric secretion quantification

Female Wistar rats (*n* = 6 per group; total *n* = 30) were distributed into experimental groups, weighed one day prior to the experiment, and fasted for 18 h. Animals were anesthetized with ketamine/xylazine (100/10 mg/kg, i.p.), and the abdominal region was shaved.

An epigastric incision (∼1.5 cm) exposed the stomach, followed by pylorus ligation. Treatments were administered intraduodenally: vehicle (saline, 10 mL/kg), positive control (cimetidine, 100 mg/kg, Merck^®^—Cat. No. C4522), *F. chica* (135 mg/kg), TRF (15 mg/kg), and *F. chica* + TRF (ED_50_ = 150 mg/kg). Euthanasia was performed four hours after abdominal cavity closure.

Stomachs were removed, washed externally with distilled water, and dried with gauze. Gastric contents were collected after internal washing with distilled water (2 mL) and centrifuged (2500 rpm, 10 min). The supernatant was used to determine volume, pH, and total acidity.

Volume was adjusted to 10 mL with distilled water, and pH was determined using a pH meter. Total acidity was quantified by titration with 0.05 N (LS Chemicals, Brazil; Cat. No. HS0005) and expressed as mEq[H^+^]/mL/4 h (Figueiredo et al. 2023).

### Acetic acid-induced ulcer

Female Wistar rats (*n* = 20) were divided into experimental groups (*n* = 4 per group), weighed one day before the experiment, and fasted for 18 h. On the day of the procedure, animals were anesthetized with ketamine/xylazine (100/10 mg/kg, i.p.).

A midline laparotomy was performed in the epigastric region, exposing the stomach. A filter paper disk (6 mm diameter) soaked in glacial acetic acid (Labsynth^®^—Cat. No.A1019.01.BJ) was applied to the gastric serosal surface for 90 s. Thereafter, with removal of the paper, the area was washed with saline solution, the stomach was repositioned, and the abdominal cavity was sutured (Wang et al. 2017).

Three days after ulcer induction, animals were treated orally (once daily for 7 days) with vehicle (saline, 2.5 mL/kg), positive control (cimetidine, 100 mg/kg, Merck^®^—Cat. No. C4522), *F. chica* (135 mg/kg), TRF (15 mg/kg), or *F. chica* + TRF (ED_50_ = 150 mg/kg). Euthanasia was performed on the eighth day.

After removal, the stomachs were opened along the greater curvature and rinsed with 0.9% saline solution. The tissues were placed between glass plates and gently pressed for imaging. The ulcerated area was determined using ImageJ^®^ software. Gastric tissue fragments from the ulcerated region were collected for histological analysis using hematoxylin and eosin (H&E) and Masson’s trichrome (MT) staining.

### Histopathological analyses

Histopathological analysis was performed using gastric tissue fragments obtained from both the ethanol-induced acute gastric ulcer model (*n* = 3 animals/group) and the acetic acid-induced chronic gastric ulcer model (*n* = 3 animals/group). Fragments containing the ulcerated region were fixed in 10% formaldehyde (Êxodo^®^—Cód. No. FT07107SO) and processed for paraffin embedding. Blocks were sectioned on a microtome and stained with H&E and MT.

Slides were examined using an inverted brightfield optical microscope (Leica DMI 4000B) equipped with a digital camera, using 5× and 10× magnifications. Collagen fiber organization was evaluated by second-harmonic generation (SHG) microscopy using an LSM 780-NLO Zeiss confocal microscope (Axio Observer Z.1) equipped with a pulsed laser (100 fs, 80 MHz; Chameleon Discovery NX, Coherent Inc.). The laser was focused on the sample through a 20× magnification, and the SHG signal was collected in transmitted mode *via* a condenser (NA 0.8; WD 26 mm) and detected by a non-descanned detector (NDD) with an SP/485 filter. The two-photon excitation fluorescence (TPEF) signal was collected in reflected mode by an NDD detector.

Histopathological evaluation was performed jointly with an independent researcher experienced in histopathology who was not involved in the study. Histological findings were interpreted according to previously published histopathological criteria for experimental gastric ulcer models.

### Inflammatory cytokine quantification

Gastric tissue samples (*n* = 4/group) obtained from the acetic acid-induced gastric ulcer model were dissected on ice, weighed, washed with cold phosphate-buffered saline (PBS; Nova Biotecnologia, Brazil; Cat. No. 13-30260-05), and dried. Tissue was homogenized in 1× RIPA buffer (Merck^®^—Cat. No. 20–188) supplemented with a protease inhibitor (Merck^®^—Cat. No. 539131) at a ratio of 120 µL/mg of tissue using a homogenizer (Ultra-Turrax T10 basic, IKA^®^).

Homogenates were kept under agitation for 2 h and then centrifuged at 4 °C (14,000 rpm, 20 min). The supernatant was collected for analysis and the pellet discarded. The inflammatory cytokines IL-10, TNF-α, and IL-1β were quantified using a commercial MILLIPLEX MAP Rat Cytokine/Chemokine Magnetic Bead Panel—Immunology Multiplex Assay (Merck^®^—Cat. No. RECYTMAG-65K).

### Statistical analysis

Results were expressed as mean ± standard error of the mean (SEM). For the *in vitro* cell viability assays, two independent experiments were performed, with each concentration evaluated in technical triplicate within each experiment. Technical replicates were averaged within each independent experiment before statistical analysis, and the independent experiments were considered the experimental replicates. Comparisons of cell viability between the 5% and 0.2% FBS conditions were performed using two-way analysis of variance (ANOVA) followed by Bonferroni’s multiple-comparison test. For the *in vivo* studies, each animal was considered an independent experimental unit (biological replicate). Data were analyzed using one-way ANOVA followed by Dunnett’s multiple-comparison test, with the vehicle group used as the reference for the primary comparisons. Additional comparisons among treatment groups were performed using Tukey’s multiple-comparison test and are presented in the Supplementary Material. Statistical significance was set at *p* < 0.05. Statistical analyses were performed using Prism^®^ 5.0 (GraphPad Software, Inc., San Diego, CA, USA).

## Results

### Chemical characterization of 3-deoxiantocyanidins

Qualitative HPLC–UV/Vis analysis confirmed the presence of the characteristic 3-deoxyanthocyanidins of *Fridericia chica*: 3′-hydroxy-carajurone (6,7,3′,4′-tetrahydroxy-5-methoxyflavylium), carajurone (6,7,4′-trihydroxy-5-methoxyflavylium), and carajurin (6,7-dihydroxy-5,4′-dimethoxyflavylium) (Figure 1(A)). Apigenin and luteolin were also identified as additional phytochemical markers in 345 nm (Figure 1(B)). These results are consistent with previously reported chromatographic profiles from our research group for *F. chica* extracts (Figueira et al. 2010; Taffarello et al. 2013; Servat-Medina et al. 2015).

**Figure 1.**
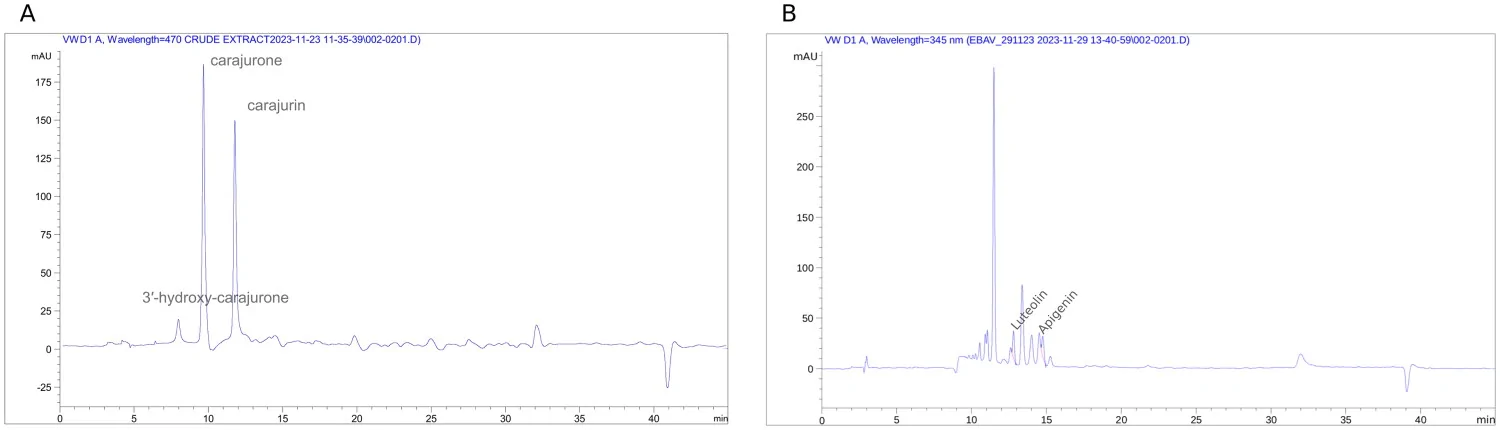
HPLC–UV/Vis chromatographic profiles of *Fridericia chica* standardized crude extract. (A) Chromatogram recorded at 470 nm showing the characteristic 3-deoxyanthocyanidins: (1) 3′-hydroxy-carajurone (6,7,3′,4′-tetrahydroxy-5-methoxyflavylium; Rt = 7.8 min); (2) carajurone (6,7,4′-trihydroxy-5-methoxyflavylium; Rt = 9.4 min); and (3) carajurin (6,7-dihydroxy-5,4′-dimethoxyflavylium; Rt = 11.6 min). (B) Chromatogram recorded at 375 nm showing the flavones apigenin (Rt = 14.6 min) and luteolin (Rt = 12.8 min).

Quantitative standardization of the extract was performed using isolated carajurin as the analytical reference standard, whereas commercial standards were employed for apigenin and luteolin. The contents of these phytochemical markers in the extract are presented in Table 1.

**Table 1.** Quantitative analysis of carajurin, apigenin, and luteolin in the standardized *Fridericia chica* extract.

Compound	Retention time (min)	Content (%w/w)	CV (%)
Carajurin	11.6	4.61 ± 0.27	5.93
Apigenin	14.6	0.21 ± 0.02	9.11
Luteolin	12.8	0.17 ± 0.001	2.61

Data are expressed as mean ± standard deviation (SD) of three independently prepared sample solutions.

CV: coefficient of variation.

The quantitative analysis confirmed the presence and content of the principal phytochemical markers in the standardized extract, with carajurin used as the principal chemical marker of *F. chica*. In addition, apigenin and luteolin were quantified, further supporting the chemical characterization of the extract used throughout the biological assays. These data provide quantitative support for the phytochemical standardization of the extract employed in the present study.

### *In vitro* pharmacological evaluation

Figure 2(A) shows the viability of HaCaT cells after exposure to *F. chica* standardized crude extract (SCE). In medium supplemented with 5% FBS, a slight increase in cell viability was observed up to 6.25 µg/mL. Under growth-restricted conditions (0.2% FBS), viability ranged from 75% to 100%. No statistically significant differences in cell viability were observed between the 0.2% and 5% FBS conditions at any of the evaluated concentrations (*p* > 0.05). In both conditions, concentrations of 50 and 100 µg/mL exhibited cytotoxic effects.

**Figure 2.**
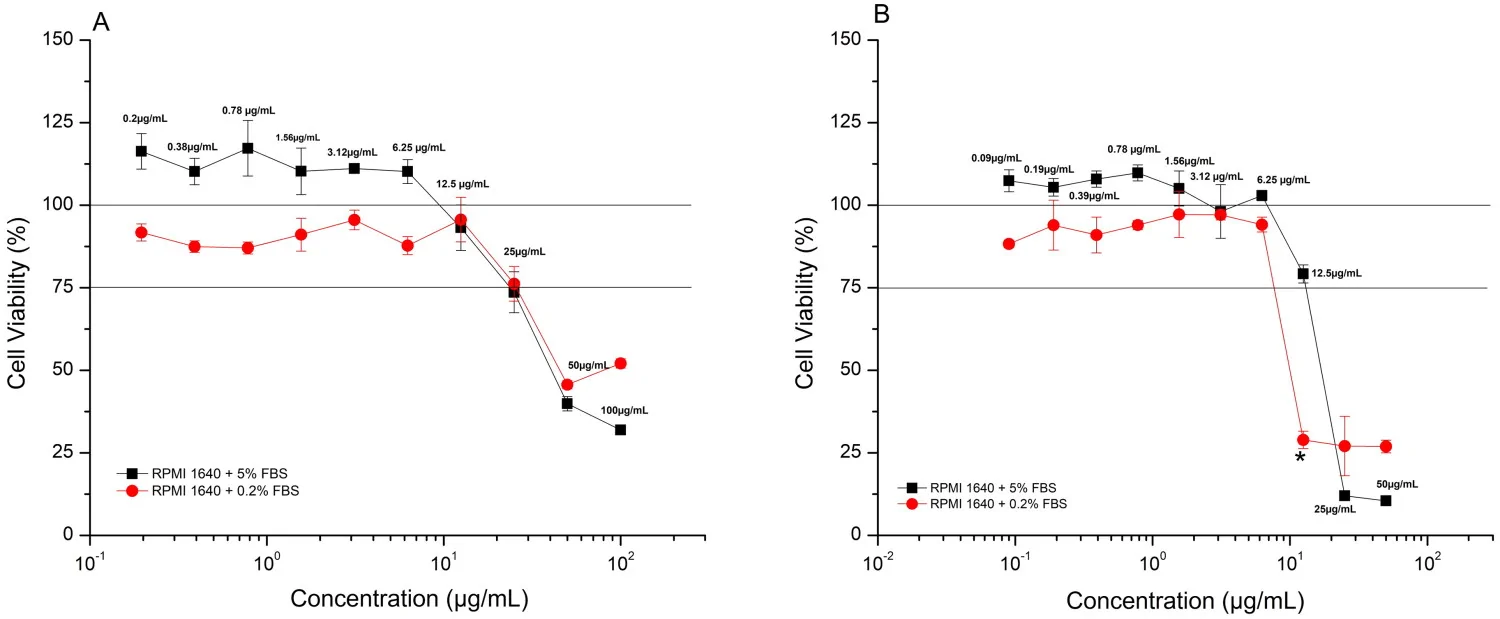
Cell viability of HaCaT cells treated with *F. chica* (A) and TRF (B) in RPMI 1640 medium containing 5% and 0.2% FBS. Data are derived from two independent experiments, with each concentration evaluated in technical triplicate within each experiment. Technical replicates were averaged within each independent experiment before statistical analysis. Statistical comparisons were performed using two-way ANOVA followed by Bonferroni’s multiple-comparison test. *p < 0.05 indicates a significant difference between the 5% and 0.2% FBS conditions.

Figure 2(B) shows the viability of HaCaT cells following exposure to TRF. In medium containing 5% FBS, viability remained above 100% up to 6.25 µg/mL and between 75% and 100% at 12.5 µg/mL. At 25 and 50 µg/mL, viability decreased to below 25%. Under growth-restricted conditions (0.2% FBS), viability was reduced to approximately 25% starting at 12.5 µg/mL, with a statistically significant difference between the 0.2% and 5% FBS conditions observed at 12.5 µg/mL (*p* < 0.05). Under normal growth conditions (5% FBS), IC_50_ values were determined in HaCaT cells, yielding 47.8 µg/mL for *F. chica* and 16.6 µg/mL for TRF.

Based on these data, the effects of *F. chica*, TRF, and their combinations on cell viability were evaluated in HaCaT and 3T3 cells at concentrations of 3, 10, 30, and 100 µg/mL using different ratios of *F. chica* and TRF (90:10, 95:5, and 99:1). As shown in Figure 3, the resulting IC_50_ values for HaCaT cells (Figure 3(A)) were 23.49 µg/mL for *F. chica* and 47.75 µg/mL for TRF, while those for 3T3 cells (Figure 3(B)) were 80.45 µg/mL for *F. chica* and 63.99 µg/mL for TRF.

**Figure 3.**
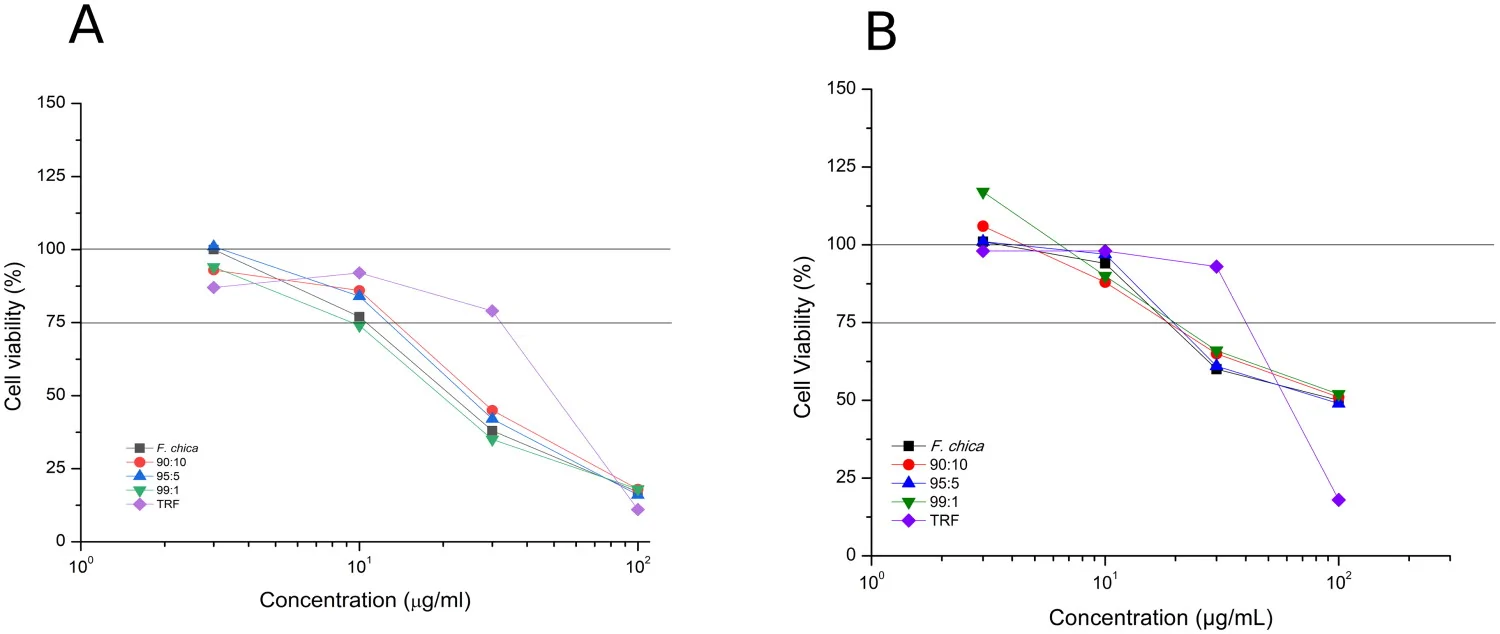
Effect of the *F. chica* and TRF combination on the viability of HaCaT (A) and 3T3 (B) cell lines. Data are derived from two independent experiments, with each concentration evaluated in technical triplicate.

These values were used to construct isobolograms (Figure 4) and evaluate the interactions between the extracts. At the tested ratios, the combinations did not markedly reduce cell viability in either cell line. The 90:10 and 99:1 ratios exhibited the lowest cytotoxicity, maintaining cell viability above 50%. Based on these results, the 90:10 ratio was selected for subsequent *in vivo* studies.

**Figure 4.**
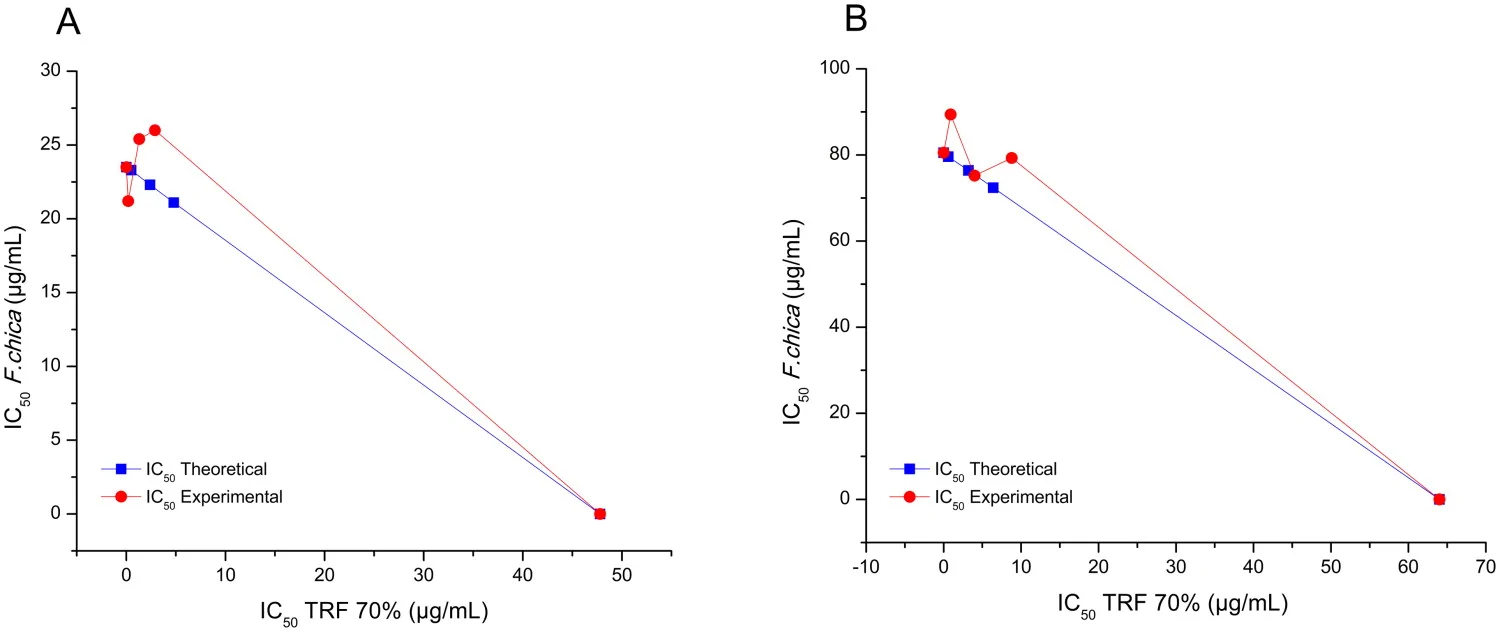
Isobologram of the effects of *F. chica* and TRF on HaCaT (A) and 3T3 (B) cell lines. Results of two independent assays, with three technical replicates per concentration.

### Dose-response curve

Administration of the *F. chica* + TRF (90:10) combination at doses of 30, 100, and 300 mg/kg was evaluated in female Wistar rats (*n* = 6/group). As shown in Figure 5, the dose–response relationship did not follow a linear pattern, with relative ulceration varying across the evaluated doses. A second-order polynomial interpolation was used as an exploratory approach to describe the observed dose–response pattern and indicated that a dose of approximately 150 mg/kg corresponded to 50% relative ulceration compared with the vehicle group. Given that only three experimental dose levels were evaluated, this value should be interpreted as an approximate estimate rather than a formally established ED_50_. Based on this exploratory analysis, 150 mg/kg was selected for subsequent experimental evaluation. Because the interpolation was based on three experimental dose levels, the fitted polynomial mathematically passed through all experimental points, resulting in an R^2^ value of 1. Therefore, this R^2^ value reflects the mathematical characteristics of the interpolation rather than the absence of biological variability.

**Figure 5.**
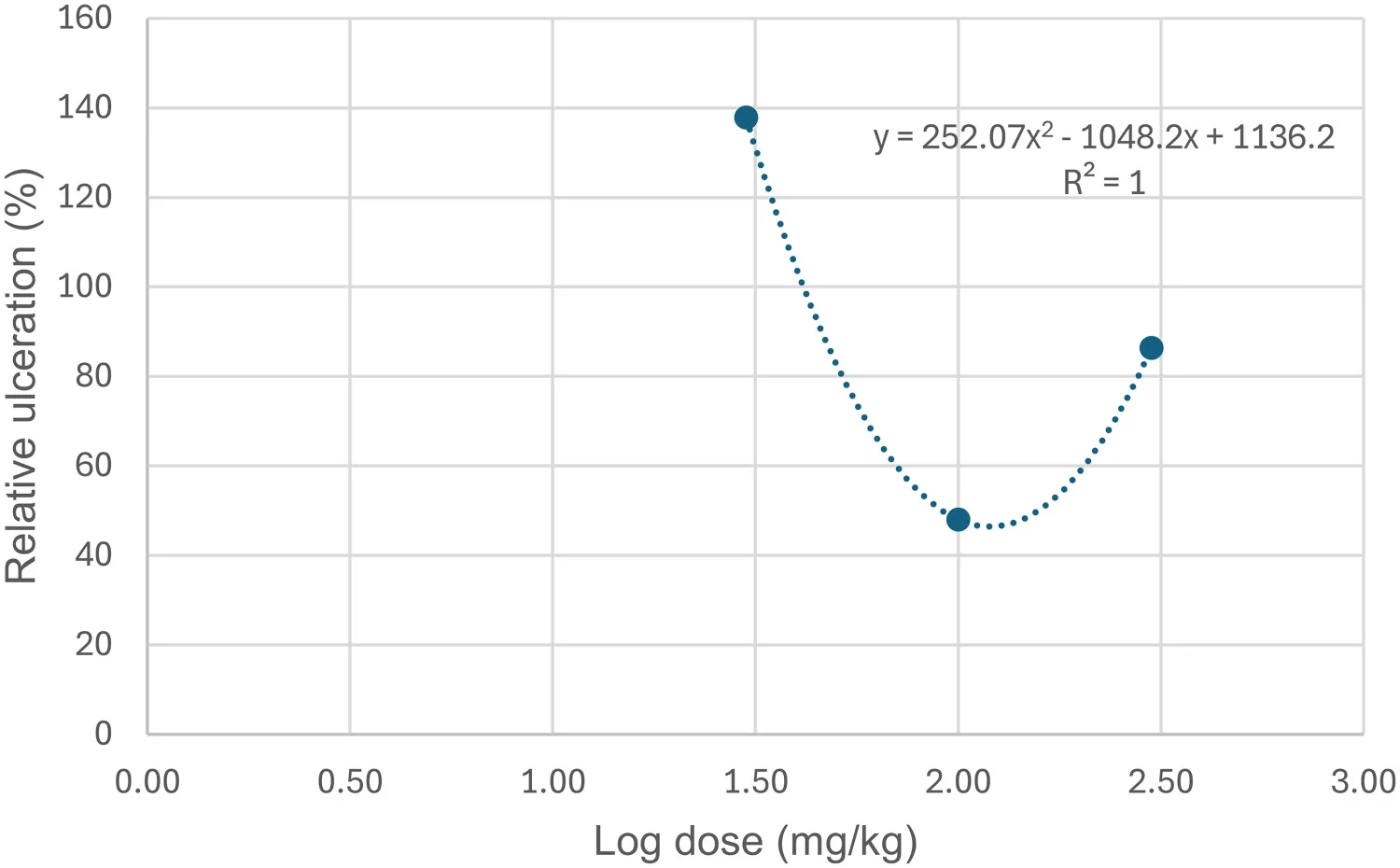
Dose–response relationship for the *F. chica* + TRF combination at doses of 30, 100, and 300 mg/kg in the ethanol-induced gastric ulcer model (*n* = 6 animals per group). Relative ulceration (%) was calculated as the percentage of ulceration in each treated group divided by the percentage of ulceration in the vehicle group × 100.

### Effect of treatments on acute ethanol-induced ulcer

After determining the ED_50_ (150 mg/kg) of the combination, experiments were conducted in an ethanol-induced gastric ulcer model in female Wistar rats (*n* = 6/group) treated with vehicle (saline + 0.2% CMC), positive control (carbenoxolone, 200 mg/kg), *F. chica* (135 mg/kg), TRF (15 mg/kg), or the *F. chica* + TRF combination (150 mg/kg).

All treatments reduced the ulcerated area compared to the vehicle group (Figure 6(A)). Healing percentages were 67.9% (*F. chica*), 66.8% (TRF), 82.8% (carbenoxolone), and 76.5% (*F. chica* + TRF) (Figure 6(B)). Representative images of the stomachs are presented in Figure 7.

**Figure 6.**
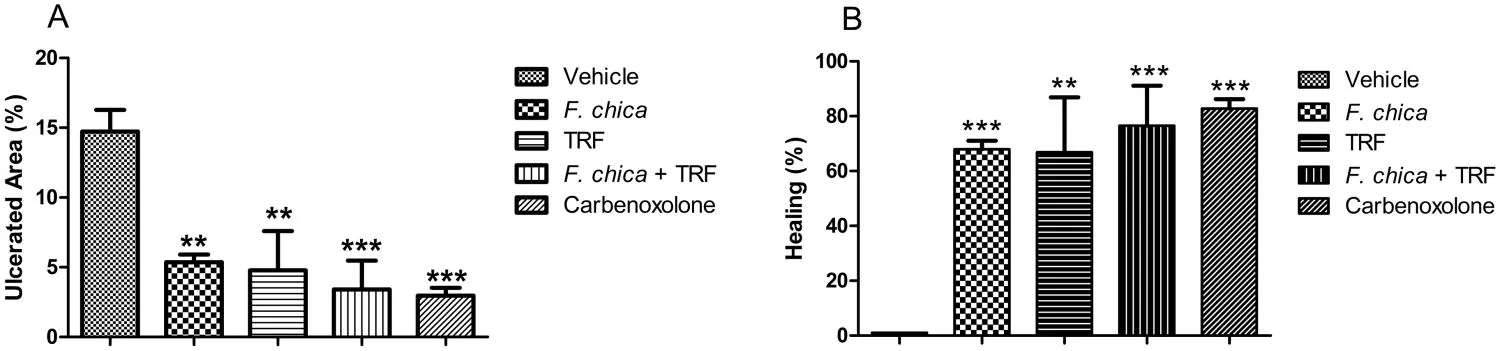
Percentage of ulcerated area and percentage of healing in the ethanol-induced gastric ulcer model (*n* = 6/group) after the respective treatments. Vehicle: saline + 0.2% CMC (2.5 mL/kg); carbenoxolone: positive control (200 mg/kg); *F. chica* (135 mg/kg); TRF: tocotrienol-enriched fraction (15 mg/kg); *F. chica* + TRF (150 mg/kg). Results are expressed as mean ± SEM. Statistical analysis was performed using one-way ANOVA followed by Dunnett’s multiple comparison test for comparisons with the vehicle group. Additional pairwise comparisons were performed using Tukey’s multiple comparison test and are presented in Supplementary Table S1. **p < 0.01; ***p < 0.001 vs. vehicle group.

**Figure 7.**
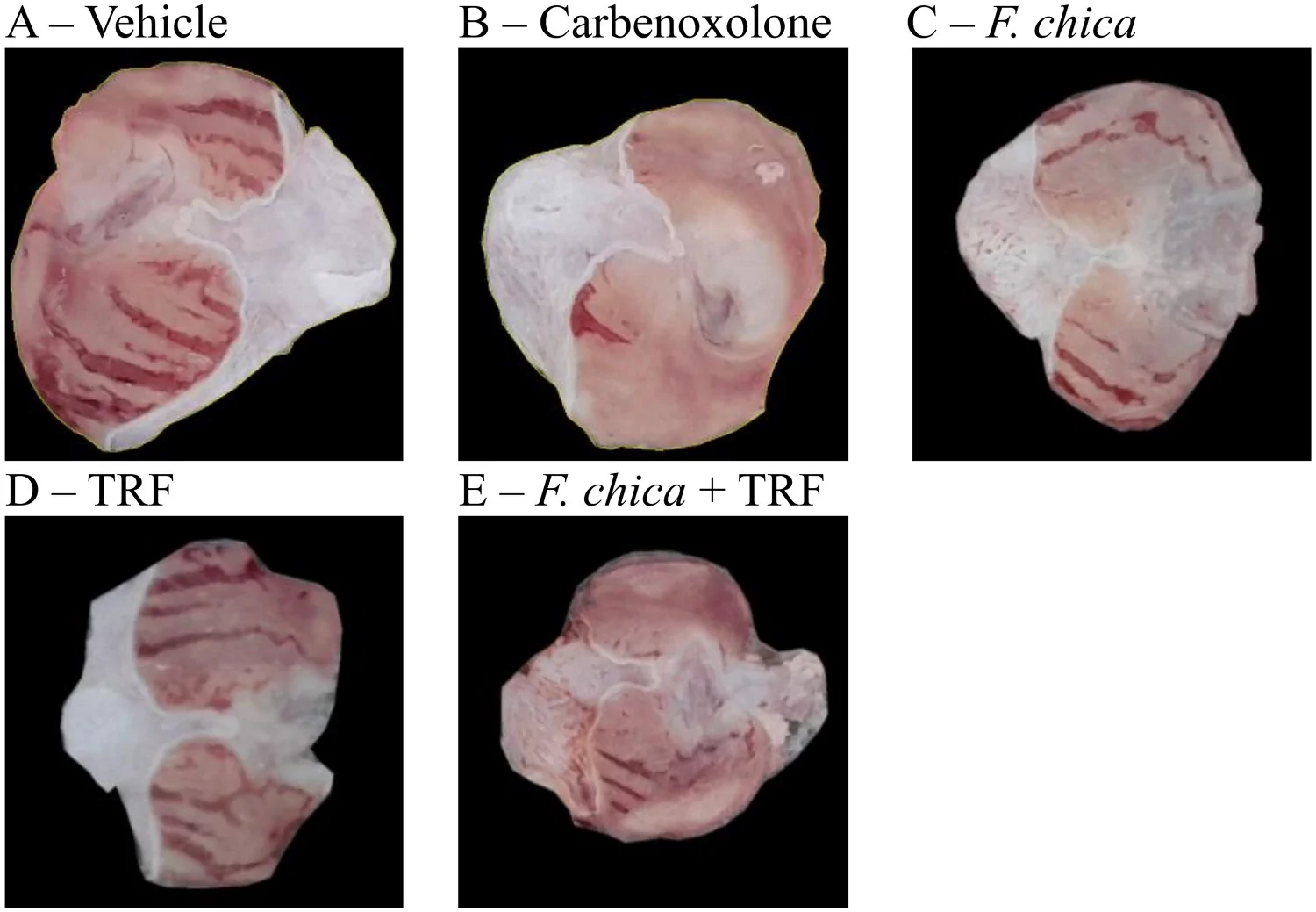
Representative gastric images from the acute ethanol-induced gastric ulcer model (*n* = 6/group) after treatments (A–E). A: vehicle—saline + 0.2% CMC (2.5 mL/kg); B: carbenoxolone—positive control (200 mg/kg); C: *F. chica* extract (135 mg/kg); D: TRF—tocotrienol-enriched fraction (15 mg/kg); E: *F. chica* + TRF combination (150 mg/kg).

### Gastric mucus quantification

After ethanol ulcer induction, mucus adhered to the gastric wall was quantified (*n* = 6/group). Treatments with carbenoxolone (200 mg/kg) and *F. chica* + TRF showed a statistically significant increase in gastric mucus content (*p < 0.05), with values of 10.28 ± 1.08 and 10.65 ± 1.37 µg of alcian blue/g of tissue, respectively, compared to the vehicle group (1.26 ± 0.12 µg/g). Treatments with isolated *F. chica* and TRF yielded values of 6.46 ± 0.61 µg/g and 9.02 ± 1.21 µg/g, respectively (Figure 8).

**Figure 8.**
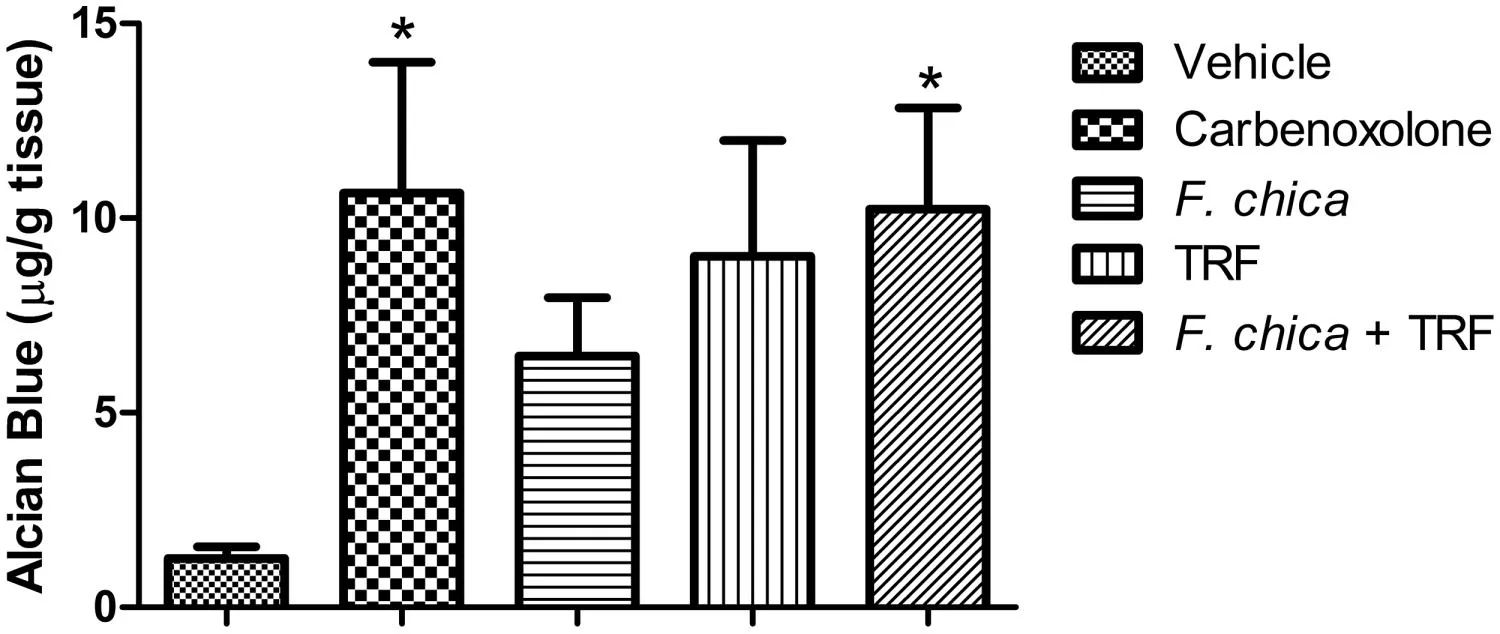
Gastric mucus quantification in the acute ethanol-induced gastric ulcer model (*n* = 6/group). Vehicle: saline + 0.2% CMC (2.5 mL/kg); carbenoxolone: positive control (200 mg/kg); *F. chica* (135 mg/kg); TRF: tocotrienol-enriched fraction (15 mg/kg); *F. chica* + TRF (150 mg/kg). Results expressed as mean ± SEM. One-way ANOVA followed by Dunnett’s test. *p < 0.05 vs. vehicle group.

### Stomach acidity evaluation

To assess gastric acid secretion, pylorus ligation was performed, followed by intraduodenal administration of treatments. According to the results obtained (Table 2), with the exception of cimetidine (100 mg/kg) and TRF (15 mg/kg), no significant differences in pH change or total acidity were observed for the other treatments compared to the vehicle.

**Table 2.** Effect of treatments on gastric acid secretion in the pylorus ligation model in rats.

Treatment	Gastric secretion volume (mL)	pH	Total acidity (mEq[H+]/mL/4 h)
Vehicle (saline + 0.2% CMC)	2.48 ± 0.12	4.14 ± 0.14	0.0140 ± 0.0020
Positive control (cimetidine)	2.73 ± 0.08	5.70 ± 0.64*	0.0070 ± 0.0004**
*F. chica*	2.30 ± 0.04	3.57 ± 0.06	0.0120 ± 0.0009
TRF	2.22 ± 0.05	3.49 ± 0.09	0.0083 ± 0.0011*
*F. chica* + TRF	2.32 ± 0.09	4.42 ± 0.26	0.0111 ± 0.0020

Results expressed as mean ± SEM. One-way ANOVA followed by Dunnett’s test.

*p < 0.05; **p < 0.01 vs. vehicle group (saline + 0.2% CMC).

### Histopathological analysis of acute ulcer

To evaluate the healing process in acute ulcer, histological sections of ulcerated areas were prepared and stained with H&E and MT. Collagen fiber organization was also qualitatively evaluated by second-harmonic generation (SHG) microscopy (Figure 9).

**Figure 9.**
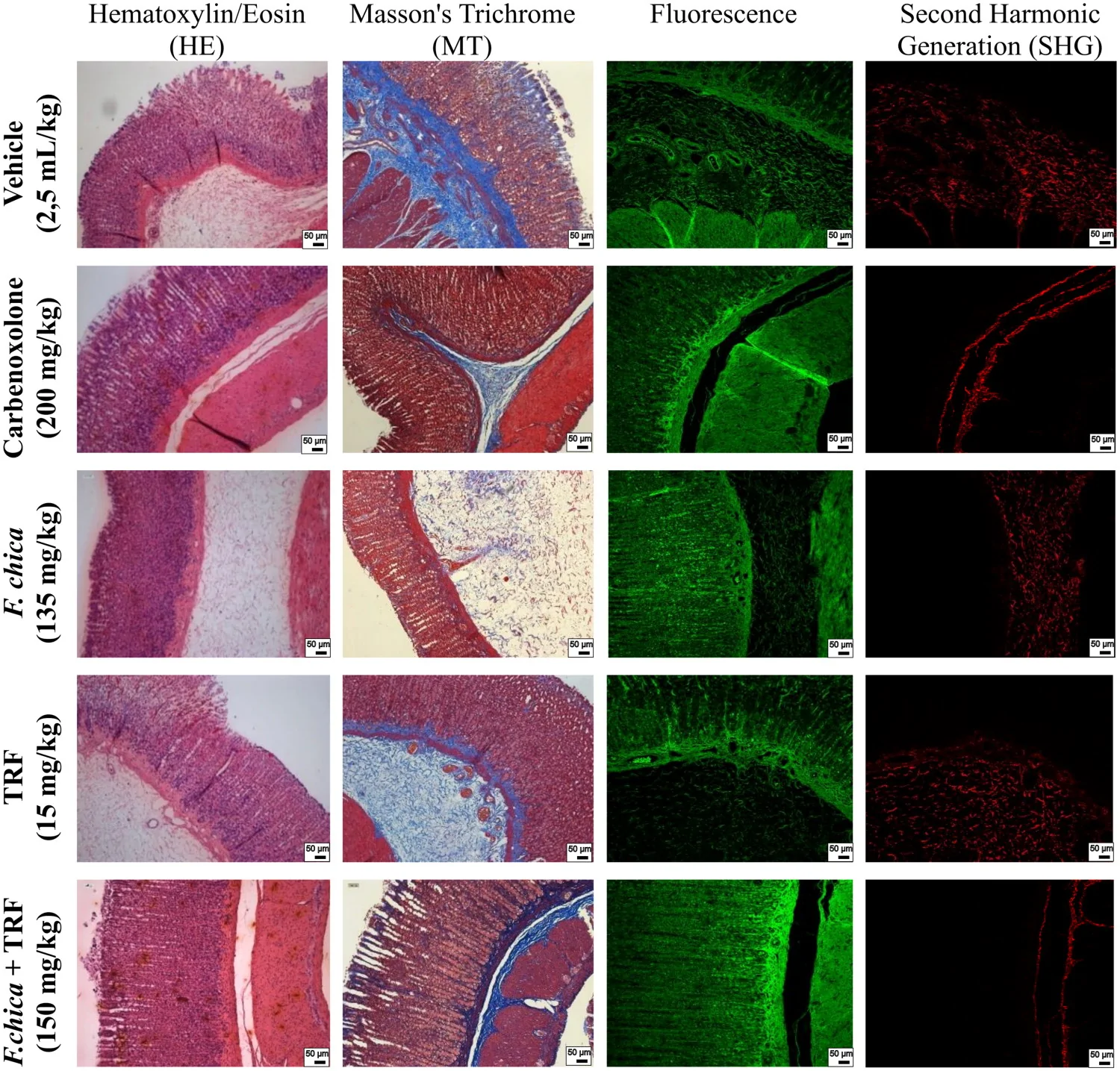
Histopathology of ethanol-induced gastric ulcer model (*n* = 3/group) after treatments, stained with H&E and MT and visualized by second-harmonic generation fluorescence microscopy. H&E: hematoxylin and eosin; MT: Masson’s trichrome. Treatments: vehicle (saline + 0.2% CMC, 2.5 mL/kg); carbenoxolone—positive control (200 mg/kg); *F. chica* (135 mg/kg); TRF: tocotrienol-enriched fraction (15 mg/kg); *F. chica* + TRF (150 mg/kg). Scale bars = 50 µm.

In animals treated with vehicle (saline + 0.2% CMC), extensive lesions were observed in the gastric mucosa, characterized by desquamation, widening of the gastric pits, the presence of blood cells, inflammatory infiltrate, and submucosal edema. MT staining revealed collagen deposition in the lamina propria, submucosa, and muscular layer, with poorly organized fibers predominating in the submucosa.

The carbenoxolone group (200 mg/kg) exhibited a preserved mucosa with discrete inflammatory infiltration and reduced submucosal edema. Collagen deposition was minimal and organized, in a pattern similar to that of healthy tissue.

In the *F. chica* (135 mg/kg) treatment group, mucosal preservation was observed along with a reduction in inflammatory infiltrate and blood cells relative to the vehicle group. However, submucosal edema persisted, with poorly organized collagen deposition in that region.

TRF (15 mg/kg) also presented a preserved mucosa, with collagen deposition similar to that observed for *F. chica*, concentrated in the submucosal region with low fiber organization.

The *F. chica* + TRF combination (150 mg/kg) showed an intact and thickened mucosa without submucosal edema. Collagen fibers were arranged in an organized manner along the submucosa, as observed in the SHG images, in a pattern similar to the positive control group.

### Effect of treatments on chronic acetic acid-induced ulcer

To evaluate the healing potential, the chronic acetic acid-induced ulcer model was used in female Wistar rats (*n* = 4/group). Three days after induction, animals were treated orally (once daily for 7 days) with vehicle (saline + 0.2% CMC), positive control (cimetidine, 100 mg/kg), *F. chica* (135 mg/kg), TRF (15 mg/kg), or the *F. chica* + TRF combination (150 mg/kg).

Body weight changed significantly over the seven-day treatment period (*p* < 0.0001); however, no significant effect of treatment (*p* = 0.5153) or treatment × time interaction (*p* = 0.4929) was observed, indicating that body weight changes were not treatment-dependent (Figure S1). In addition, no overt treatment-related clinical signs or signs of distress were observed during the treatment period.

All treatments reduced the ulcerated area compared to the vehicle group (Figure 10). The vehicle group presented an area of 12.33 ± 1.45 mm^2^, whereas the cimetidine and *F. chica* + TRF groups showed the smallest areas: 3.00 ± 0.40 mm^2^ and 1.97 ± 0.03 mm^2^, respectively.

**Figure 10.**
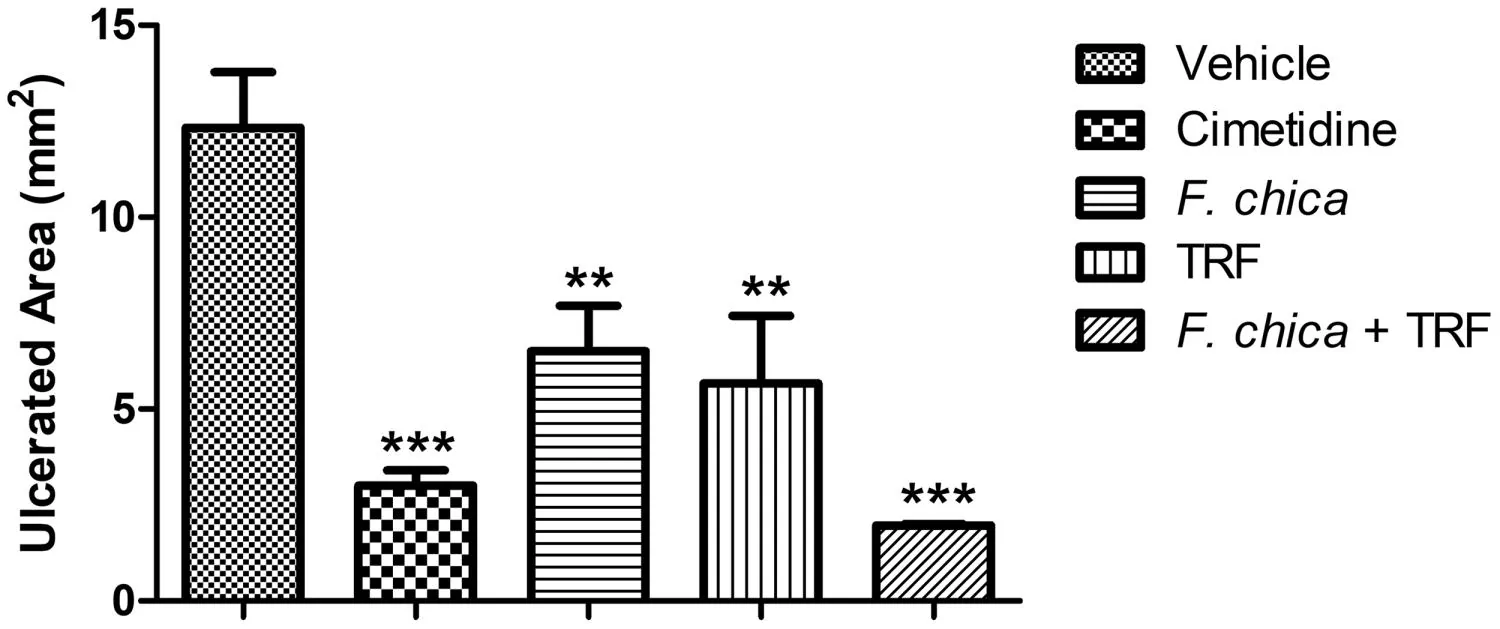
Ulcerated area (mm^2^) in the chronic acetic acid-induced gastric ulcer model (*n* = 4/group) after the respective treatments. Vehicle: saline + 0.2% CMC (2.5 mL/kg); cimetidine: positive control (100 mg/kg); *F. chica* (135 mg/kg); TRF: tocotrienol-enriched fraction (15 mg/kg); *F. chica* + TRF (150 mg/kg). Results are expressed as mean ± SEM. Statistical analysis was performed using one-way ANOVA followed by Dunnett’s multiple comparison test for comparisons with the vehicle group. Additional pairwise comparisons were performed using Tukey’s multiple comparison test and are presented in Supplementary Table S1. **p < 0.01; ***p < 0.001 vs. vehicle group.

Treatments with isolated *F. chica* and TRF yielded ulcerated areas of 6.50 ± 1.19 mm^2^ and 5.67 ± 1.76 mm^2^, respectively. Representative images of the stomachs post-treatment are presented in Figure 11.

**Figure 11.**
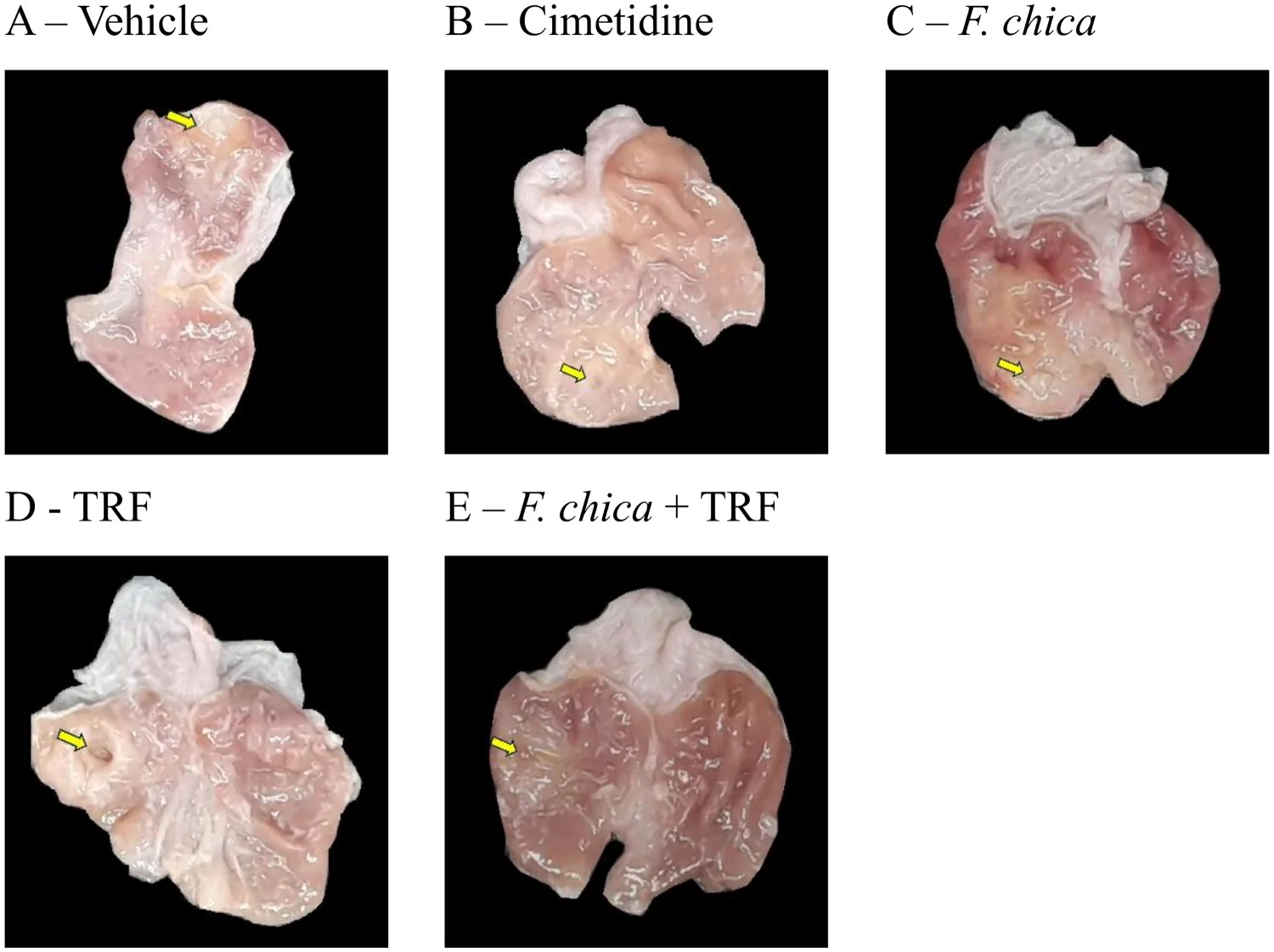
Representative gastric images from the chronic acetic acid-induced gastric ulcer model (*n* = 4/group) after treatments (a–E) administered for 7 consecutive days. A: vehicle (saline + 0.2% CMC, 2.5 mL/kg); B: cimetidine—positive control (100 mg/kg); C: *F. chica* extract (135 mg/kg); D: TRF—tocotrienol-enriched fraction (15 mg/kg); E: *F. chica* + TRF combination (150 mg/kg).

### Histopathological analysis of chronic ulcer

Histological evaluation of chronic acetic acid-induced ulcers was performed using H&E and MT staining, with qualitative analysis of collagen fibers by SHG microscopy (Figure 12).

**Figure 12.**
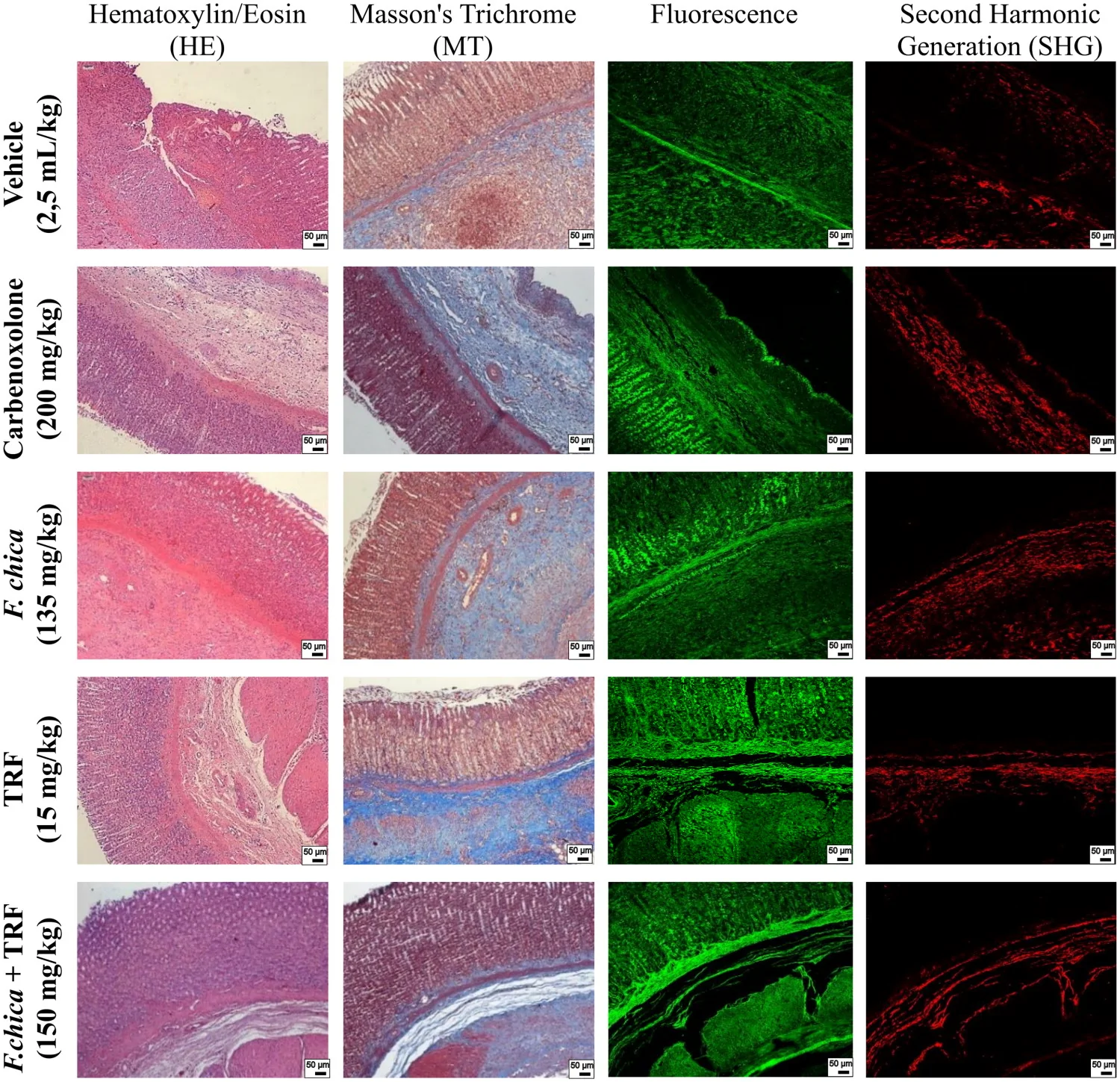
Histopathology of acetic acid-induced gastric ulcers (*n* = 3/group) after treatments, stained with H&E and MT and visualized by second-harmonic generation fluorescence microscopy. H&E: hematoxylin and eosin; MT: Masson’s trichrome. Treatments: vehicle (saline + 0.2% CMC, 2.5 mL/kg); cimetidine—positive control (100 mg/kg); *F. chica* (135 mg/kg); TRF: tocotrienol-enriched fraction (15 mg/kg); *F. chica* + TRF (150 mg/kg). Scale bars = 50 µm.

In animals treated with vehicle (saline + 0.2% CMC), extensive damage to the gastric mucosa was observed, with disorganization of the layers, inflammatory infiltrate, loss of delineation between the submucosal and muscular layers, and degenerated parietal and chief cells. Collagen deposition was diffuse in the lesioned area, with disorganized fibers as seen in the MT and SHG images.

The cimetidine group (100 mg/kg) showed partial mucosal preservation with granulation tissue and neovascularization in the submucosa, alongside inflammatory infiltrate and areas of damage in the muscular layer. Collagen deposition was noted in the lamina propria and in the granulation tissue region, displaying greater organization than the vehicle group.

Treatments with *F. chica*, TRF, and the *F. chica* + TRF combination all showed mucosal preservation with vacuolated parietal cells and widened gastric pits. Areas with granulation tissue and neovascularization were observed, as were regions with better-defined gastric wall layers. Collagen deposition was evident in granulation tissue areas, while in regions with higher tissue organization, fibers displayed a more defined arrangement along the submucosa. In the *F. chica* + TRF combination group, areas with preserved tissue architecture were also observed.

### Inflammatory cytokine quantification in chronic ulcer

The cytokines TNF-α, IL-1β, and IL-10 were quantified in ulcerated gastric tissue (*n* = 4/group) after acetic acid induction and 7 days of treatment, with euthanasia on the eighth day.

A significant reduction in TNF-α levels (*p* < 0.05) was observed in the groups treated with TRF and the *F. chica* + TRF combination (Figure 13(A)).

**Figure 13.**
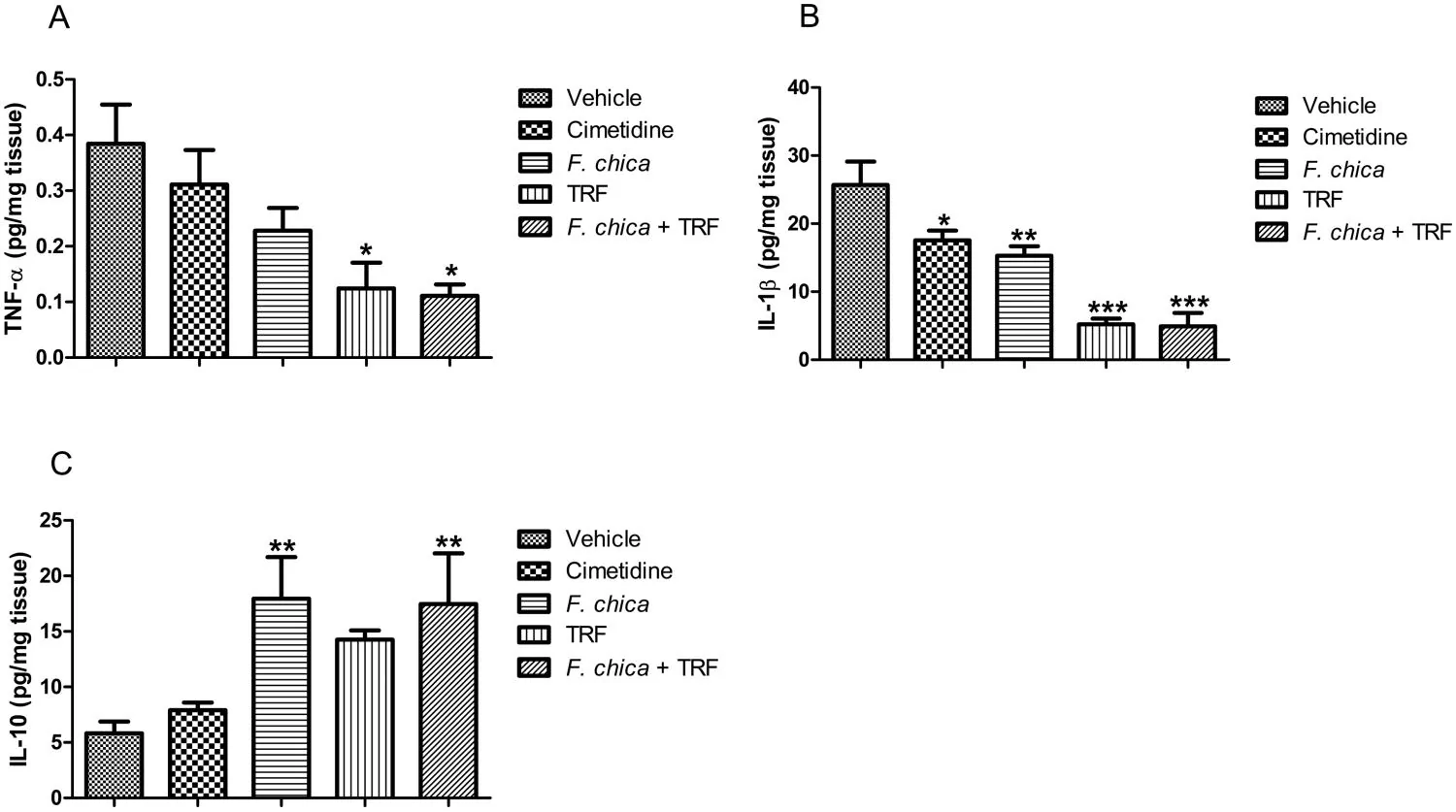
Quantification of pro-inflammatory cytokines (TNF-α and IL-1β) and anti-inflammatory cytokine IL-10 in chronic acetic acid-induced ulcers (*n* = 4/group) in rats. Vehicle: saline + 0.2% CMC (2.5 mL/kg); cimetidine: positive control (100 mg/kg); *F. chica* (135 mg/kg); TRF: tocotrienol-enriched fraction (15 mg/kg); *F. chica* + TRF (150 mg/kg). Results expressed as mean ± SEM. One-way ANOVA followed by Dunnett’s test. *p < 0.05; **p < 0.01; ***p < 0.001 vs. vehicle group.

For IL-1β (Figure 13(B)), all treatments promoted a significant reduction compared to the vehicle group. The cimetidine and *F. chica* groups showed reductions of 31.8% (*p* < 0.05) and 40.5% (*p < 0.01), respectively, while TRF and the combination presented reductions of 79.7% and 80.9% (*p* < 0.001), respectively.

IL-10 quantification (Figure 13(C)) demonstrated a significant increase in the groups treated with *F. chica* and the combination (*p* < 0.01), with elevations of 67.9% and 66.6%, respectively. The cimetidine and TRF groups showed increases of 26.37% and 59.1%, respectively, compared to the vehicle.

## Discussion

The choice of cell lines for cytotoxicity evaluation must consider the stage of the healing process under investigation. Keratinocytes and fibroblasts are widely used in this context, as they represent the primary cells involved in the proliferative phase of cutaneous healing (Guerrero et al. 2024).

Cell viability assay on the HaCaT cell line showed that both the standardized crude extract (SCE) of *F. chica* and the tocotrienol-enriched fraction (TRF) exhibited cytotoxic effects at the highest concentrations tested. This effect may be associated with the antioxidant potential of the extracts, particularly TRF, which exhibits high antioxidant activity (DPPH assay: IC_50_ = 123.64 ± 25.54 µg/mL), as observed in studies by our research group.

The cytotoxic effect noted for *F. chica* is consistent with the findings of Zago (2014), who, using human keratinocyte cell lines, demonstrated increased cell viability at concentrations of 5 and 10 µg/mL (112.2% and 105.4%, respectively), whereas the 50 µg/mL concentration exhibited a cytotoxic effect. This behavior may be associated with the plant’s complex phytochemical matrix, where multiple constituents may exert individual and complementary biological actions, ultimately giving rise to concentration-dependent effects. Nevertheless, literature reports the safe use of the extract both in *in vitro* studies with Chinese hamster ovary epithelial cells (Mafioleti et al. 2013) and in a phase I clinical trial using a gel containing 2.5% standardized *F. chica* extract (Jorge et al. 2020).

Although antioxidant activity is desirable in the healing process, these compounds can also generate reactive species during redox reactions. Studies show that phenolic compounds can interact with culture medium components, leading to the formation of hydrogen peroxide and other oxidation products, which may induce cytotoxicity independently of the cell line (Long and Halliwell 2011; Kelts et al. 2015).

In the biological context, reactive oxygen species participate in essential physiological processes such as phagocytosis, energy production, cell growth regulation, and intracellular signaling. However, when their production exceeds the system’s antioxidant capacity, damage to cellular macromolecules, including proteins, lipids, DNA, and carbohydrates, can occur, leading to cellular dysfunction and apoptosis (Franco et al. 2021).

Therefore, determining appropriate concentrations of antioxidant compounds is fundamental to maintaining redox balance and ensuring beneficial effects. Based on this concept, the proportion of TRF was reduced to generate the 90:10, 95:5, and 99:1 combinations. These combinations showed lower cytotoxicity under the experimental conditions evaluated, as previous assays had shown that standard 50:50, 25:75, and 75:25 combinations fell below the additivity isobole, resulting in increased cytotoxicity (data not shown). After adjusting proportions to safer values, isobolograms were constructed; in both cell lines, the 90:10 ratio emerged as the point associated with the least reduction in cell viability.

It is important to emphasize that the objective of the isobologram analysis in the present study differed from its conventional application in studies evaluating combinations of cytotoxic agents. Because the IC_50_ was used as the endpoint, the aim was not to identify a synergistic increase in cytotoxicity, but rather to select the proportion that best preserved cell viability and was suitable for subsequent *in vivo* evaluation. In this context, increased cytotoxicity would not represent a desirable outcome, since the intended application of the formulation is gastroprotection and tissue repair rather than the elimination of viable cells. Therefore, the 90:10 ratio was selected because it consistently exhibited the most favorable balance between preservation of cell viability and suitability for subsequent *in vivo* evaluation.

Extracts of *F. chica*, TRF, and their combination at the indicated ratio (90:10) were used in acute and chronic ulcer treatments in the *in vivo* models. Doses were selected based on the dose–response curve results. The dose–response curve showed a non-linear pattern, with lower gastroprotective responses observed at both the lowest and highest doses evaluated. This is consistent with the findings from clinical and preclinical *in vivo* studies reporting the appearance of erythema and contact dermatitis at higher doses of topically administered TRF, as well as biochemical alterations when administered orally (Hasan et al. 2008; Fiume et al. 2018; Teo et al. 2021).

After determining the ED_50_ of 150 mg/kg for the *F. chica* + TRF combination in the ethanol-induced ulcer model, a new experiment was conducted for comparative analysis against the vehicle group (saline + 0.2% CMC, 2.5 mL/kg).

The results demonstrated a healing effect for all treatments. Although no statistically significant differences were observed among the active treatments regarding ulcer area reduction (Table S1), the combination exhibited consistent gastroprotective activity and was further characterized through histopathological and inflammatory analyses.

When treating animals with an anthocyanin-enriched fraction obtained from the hydroethanolic extract of *F. chica* (30 or 100 mg/kg), Miorando et al. (2025) achieved acute ulcer area reductions of 65.6% and 53.4%, respectively, values similar to those observed in the present study (67.9%) for the crude extract of *F. chica* at a dose of 150 mg/kg.

There was an increase in mucus production, with statistical significance (*p* < 0.05) for the positive control (87.74% increase) and for the combination (88.17% increase), compared to the vehicle group. The isolated extracts showed increases of 80.49% for *F. chica* and 86.03% for TRF. While the differences between values are subtle, these results reinforce the potential of the extracts to stimulate mucus production.

Previous studies have suggested that the gastroprotective effects of *F. chica* may involve increased prostaglandin availability in the gastric mucosa, potentially through inhibition of 15-hydroxyprostaglandin dehydrogenase (15-PGDH), the enzyme responsible for the first step in the degradation of these molecules (Peskar 1980; Figueiredo et al. 2023; Miorando et al. 2025, 2024). The pharmacological mechanism also appears to involve a decrease in lipid peroxidation, an increase in antioxidant enzyme activity (including GSH [reduced glutathione] and SOD [superoxide dismutase]), and modulation of inflammatory cytokines (Tahir et al. 2022; Figueiredo et al. 2023; Miorando et al. 2024).

Similarly, tocotrienols have previously been associated with gastric mucosal protection through prostaglandin-related mechanisms, including increased COX-1 expression and PGE2 production (Azlina et al. 2013). They are also involved in free radical scavenging, enhancement of antioxidant enzyme activity, normalization of gastric mucosal NO through reduced iNOS expression, and modulation of inflammatory cytokines (Azlina et al. 2015). These mechanisms were not directly evaluated in the present study but may provide a pharmacological context for the observed increase in gastric mucus production.

The antisecretory assay showed no significant reduction in gastric volume, total acidity, or free acidity following treatment with *F. chica* SCE. Previous studies using different *F. chica* preparations and doses have reported variable effects on gastric secretion. Figueiredo et al. (2023) observed increased free and total acidity at some doses, with a reduction in gastric volume only at 1 mg/kg, whereas Miorando et al. (2024) reported reductions in gastric volume and total acidity using a flavonoid-enriched fraction. These differences suggest that the effects of *F. chica* on gastric secretion may depend on extract composition, dose, and experimental conditions. In the present study, the relatively high pH and low baseline acidity observed in the vehicle group may also have limited the detection of treatment-related differences.

The biological effects of anthocyanins may also be influenced by their limited and variable oral bioavailability, which depends on their physicochemical characteristics and the gastrointestinal environment. Although their absorption is generally low, anthocyanin-derived metabolites may also contribute to their biological activity (Zhang et al. 2017; Gonçalves et al. 2021; Ayvaz et al. 2023). Further pharmacokinetic characterization may help clarify the relationship between these factors and the pharmacological effects of *F. chica*.

Histopathological analysis of ethanol-induced ulcers following combination treatment showed an absence of submucosal edema and the formation of more organized collagen fibers. These qualitative morphological findings indicate preservation and organization of gastric tissue following treatment. These findings are consistent with the gastric mucus quantification results and the higher healing percentage observed for this treatment.

In the chronic acetic acid-induced ulcer model, all treatments reduced the ulcerated area; however, the combination presented the smallest area (1.97 ± 0.03 mm^2^) although this difference was not statistically significant compared with the isolated treatments (Table S1). Cimetidine also significantly reduced the ulcerated area (3.00 ± 0.40 mm^2^). Qualitative histopathological evaluation indicated differences in tissue morphology among the treatment groups, with preserved tissue architecture and defined tissue layers observed in the *F. chica* + TRF group.

In chronic wounds, the inflammatory phase becomes persistent, resulting in deficient healing. Macrophages with pro-inflammatory phenotypes are present in high numbers and exhibit a reduced capacity to phagocytose apoptotic neutrophils, sustaining an inflammatory environment rich in pro-inflammatory cytokines such as TNF-α and IL-1β (Raziyeva et al. 2021). Pro-inflammatory interleukins stimulate the recruitment and activation of neutrophils and macrophages, and their excessive secretion triggers an exaggerated inflammatory response causing tissue damage, particularly in chronic ulcers. Conversely, the expression of anti-inflammatory interleukins such as IL-10 limits inflammation and tissue damage, helping to control the immune response. IL-10 acts by balancing the inflammatory response and is critical in chronic wounds, where prolonged inflammation can cause extensive tissue damage (Al-Qahtani et al. 2024).

The balance between pro- and anti-inflammatory mediators is essential for an appropriate healing outcome, as an imbalance can result in delayed healing or keloid formation (Wong et al. 2025). Regulation of immune cells through cytokine modulation, notably by inhibiting pro-inflammatory cytokines and increasing anti-inflammatory cytokine expression, constitutes an important strategy in treating chronic wounds (Raziyeva et al. 2021).

According to the results obtained, the combination modulated the inflammatory response in the chronic ulcer model, as demonstrated by reductions in pro-inflammatory cytokines and an increase in IL-10. The response observed in the isolated treatment groups suggests that TRF may contribute predominantly to the reduction in pro-inflammatory mediators, whereas *F. chica* may contribute to the increase in IL-10.

Figueiredo et al. (2023) analyzed the same cytokines in the *F. chica* extract, observing a reduction in all evaluated cytokines. The authors suggest that the extract’s healing capacity may be related to the suppression of pro-inflammatory cytokines, independent of anti-inflammatory cytokine levels. In contrast, the present study observed an increase in IL-10, which may reflect differences in experimental conditions and suggests a possible complementary role for anti-inflammatory cytokine modulation in the healing process.

Collectively, these findings support the gastroprotective effects of the combination, including modulation of the inflammatory response, while the qualitative histopathological findings indicate preservation and organization of gastric tissue. These results reinforce the therapeutic potential of the combination as a multifactorial approach for treating gastric lesions, particularly in chronic conditions characterized by persistent inflammation and compromised tissue repair.

## Study limitations

Histopathological evaluation was conducted with the assistance of an independent researcher experienced in histopathology, although treatment allocation was not blinded during slide assessment. Histopathological and second-harmonic generation (SHG) analyses provided qualitative evidence of tissue response and collagen organization; however, standardized semi-quantitative histopathological scoring and quantitative SHG image analysis were not included in the experimental design. In addition, oxidative stress markers were not directly measured in gastric tissues; therefore, the contribution of oxidative stress modulation to the observed gastroprotective effects requires further investigation. The final sample size in the chronic ulcer model (*n* = 4 animals per group) also limited the formal assessment of distributional assumptions and should be considered when interpreting the statistical findings from this model.

## Conclusions

The combination of *F. chica* and the tocotrienol-enriched fraction exhibited a significant antiulcerogenic effect, promoting an increase in gastric mucus production, modulating the inflammatory response, and preserving the histological architecture of gastric tissue. These findings support the gastroprotective effects of the combination in both acute and chronic ulcer models. Histopathological evaluation qualitatively indicated preservation of gastric tissue architecture and organized collagen deposition following treatment with the combination.

Although classical pharmacological synergism was not demonstrated under the experimental conditions evaluated, the combination showed gastroprotective activity when used at the selected proportion, highlighting the potential of this approach for the development of therapeutic strategies based on natural products. Overall, these findings indicate that the combination exerts a multifactorial gastroprotective effect encompassing mucus preservation and the regulation of inflammatory pathways.

Future studies should further investigate the mechanisms underlying the gastroprotective effects of the combination, particularly pathways related to oxidative stress and inflammatory regulation, including Nrf2/HO-1 and NF-κB signaling, as well as the potential involvement of prostaglandins. Pharmacokinetic studies will also be important to characterize the systemic exposure and bioavailability of the components and to explore potential exposure–response relationships. In addition, longer-term studies specifically designed for toxicological assessment would contribute to a more comprehensive characterization of the systemic safety profile of the combination.

## Supplementary Material

Suplementary material.docx

## Data Availability

The data supporting the findings of this study are available in the REDU repository at https://doi.org/10.25824/redu/9CSCXW. Additional data related to this study are available from the corresponding author upon reasonable request.
