# Supplementary material for: Fridericia chica standardized extract combined with tocotrienol-enriched fraction from Bixa orellana promotes gastric mucosal protection: in vitro combination selection and in vivo evaluation
Source: Pharm Biol. 2026 Oct 24;64(1):1168–90. doi: 10.1080/13880209.2026.2751273 (PMC13700538; doi:10.1080/13880209.2026.2751273)
Supplement: Supplementary file 1 [file IPHB_A_2751273_SM8770.docx]

Table S1. Additional pairwise comparisons among treatment groups using one-way ANOVA followed by Tukey's multiple comparison test

| Experimental model | Parameter | Comparison | Mean difference | Significance | 95% CI |
| --- | --- | --- | --- | --- | --- |
| Ethanol-induced gastric ulcer model | Ulcerated area (%) | Vehicle vs *F. chica* | 9.362 | ** | 2.366 to 16.36 |
|  | Ulcerated area (%) | Vehicle vs TRF | 9.945 | ** | 2.638 to 17.25 |
|  | Ulcerated area (%) | Vehicle vs *F. chica* + TRF | 11.31 | *** | 4.315 to 18.31 |
|  | Ulcerated area (%) | Vehicle vs CBX | 11.76 | *** | 4.762 to 18.75 |
|  | Ulcerated area (%) | *F. chica* vs TRF | 0.5834 | ns | -6.412 to 7.579 |
|  | Ulcerated area (%) | *F. chica* vs *F. chica* + TRF | 1.949 | ns | -4.721 to 8.619 |
|  | Ulcerated area (%) | *F. chica* vs CBX | 2.396 | ns | -4.274 to 9.066 |
|  | Ulcerated area (%) | TRF vs *F. chica* + TRF | 1.366 | ns | -5.630 to 8.361 |
|  | Ulcerated area (%) | TRF vs CBX | 1.812 | ns | -5.183 to 8.808 |
|  | Ulcerated area (%) | *F. chica* + TRF vs CBX | 0.4467 | ns | -6.223 to 7.117 |
|  | Healing (%) | Vehicle vs *F. chica* | 6.123 | ** | -112.7 to -21.22 |
|  | Healing (%) | Vehicle vs TRF | 5.763 | ** | -113.6 to -18.06 |
|  | Healing (%) | Vehicle vs *F. chica* + TRF | 6.906 | *** | -121.3 to -29.79 |
|  | Healing (%) | Vehicle vs CBX | 7.478 | *** | -127.5 to -36.05 |
|  | Healing (%) | *F. chica* vs TRF | 0.1035 | ns | -44.61 to 46.88 |
|  | Healing (%) | *F. chica* vs *F. chica* + TRF | 0.8214 | ns | -52.18 to 35.05 |
|  | Healing (%) | *F. chica* vs CBX | 1.421 | ns | -58.44 to 28.79 |
|  | Healing (%) | TRF vs *F. chica* + TRF | 0.8867 | ns | -55.45 to 36.05 |
|  | Healing (%) | TRF vs CBX | 1.459 | ns | -61.70 to 29.79 |
|  | Healing (%) | *F. chica* + TRF vs CBX | 0.6000 | ns | -49.87 to 37.36 |
| Acetic acid-induced gastric ulcer model | Ulcerated área (mm^2^) | Vehicle vs Cimetidine | 9.333 | *** | 4.347 to 14.32 |
|  | Ulcerated área (mm^2^) | Vehicle vs *F. chica* | 5.833 | * | 0.8469 to 10.82 |
|  | Ulcerated área (mm^2^) | Vehicle vs TRF | 6.667 | * | 1.336 to 12.00 |
|  | Ulcerated área (mm^2^) | Vehicle vs *F. chica* + TRF | 10.37 | *** | 5.036 to 15.70 |
|  | Ulcerated área (mm^2^) | Cimetidine vs *F. chica* | -3.500 | ns | -8.117 to 1.117 |
|  | Ulcerated área (mm^2^) | Cimetidine vs TRF | -2.667 | ns | -7.653 to 2.320 |
|  | Ulcerated área (mm^2^) | Cimetidine vs *F. chica* + TRF | 1.033 | ns | -3.953 to 6.020 |
|  | Ulcerated área (mm^2^) | *F. chica* vs TRF | 0.8333 | ns | -4.153 to 5.820 |
|  | Ulcerated área (mm^2^) | *F. chica* vs *F. chica* + TRF | 4.533 | ns | -0.4531 to 9.520 |
|  | Ulcerated área (mm^2^) | TRF vs *F. chica* + TRF | 3.700 | ns | -1.631 to 9.031 |

Data represent additional pairwise comparisons performed using one-way ANOVA followed by Tukey's multiple comparison test. Significance levels are indicated as follows: ns, not significant; *P < 0.05; **P < 0.01; ***P < 0.001**.**

Figure S1. Body weight changes during treatment in the chronic acetic acid-induced gastric ulcer model

Body weight was monitored throughout the seven-day treatment period, and changes were calculated relative to the initial body weight of each animal. Data are expressed as percentage change from baseline (mean ± SEM; n = 4/group). Statistical analysis was performed using two-way repeated-measures ANOVA, considering treatment and time as factors. A significant effect of time was observed (p < 0.0001), whereas no significant effect of treatment (p = 0.5153) or treatment × time interaction (p = 0.4929) was detected.
